# Bound Variable Singular *They* Is Underspecified: The Case of *All* vs. *Every*

**DOI:** 10.3389/fpsyg.2022.880687

**Published:** 2022-06-02

**Authors:** Keir Moulton, Trevor Block, Holly Gendron, Dennis Storoshenko, Jesse Weir, Sara Williamson, Chung-hye Han

**Affiliations:** ^1^Department of Linguistics, University of Toronto, Toronto, ON, Canada; ^2^Department of Linguistics, Simon Fraser University, Burnaby, BC, Canada; ^3^School of Languages, Linguistics, Literatures and Cultures, University of Calgary, Calgary, AB, Canada

**Keywords:** pronoun comprehension, bound variable anaphora, singular *they*, distributivity, quantification and number

## Abstract

The goal of this article is to investigate the factors that affect the acceptability and processing of *they*. Previous research has sought to determine whether there are acceptability and processing differences between *they/themselves* with plural vs. singular antecedents, with mixed results. The studies reported here address this question using bound variable singular *they* (e.g., *Every customer claimed that they were first in line*). We asked whether bound singular *they* is sensitive to both the morphological number and the semantic distributivity of the binding quantifier phrase. We contrasted morphologically singular quantified antecedents (*every* and *each*) with plural quantified antecedents (*all*). Instead of finding an effect of number, we found an effect of semantic distributivity in acceptability, with bound singular *they* demonstrating a cline of preference toward more distributive antecedents. Neither number nor distributivity, however, registered as an effect on reading times. Rather, for all types of quantified antecedents, encountering a pronoun like *he* or *she* rather than *they* registered a processing delay, in contrast to non-quantified antecedents. Our results are most fully compatible with the view that *they* is underspecified for number properties.

## 1. Introduction

A great deal about the human sentence processor has been learned from investigating how pronouns are integrated into sentence comprehension in real-time. Pronouns require referents, and these can be sourced from the extra-linguistic context or linguistic antecedents. The form of a pronoun—encoded by number, person, and gender features—constrains its reference, something that can be detected in the earliest moments of processing (Arnold et al., 2000). Some English pronouns, however, do not bear features that easily and unambiguously determine their referents, tolerating a range of antecedents with differing features. One particularly interesting case is the English pronoun series *they/them/their* and its reflexive counterpart (*themselves/themself*). These anaphors admit several different types of grammatical antecedents, subject to differences in semantic interpretation, discourse context, and speaker variation. *They/them/their* accepts a plural antecedent (1a), and readily takes a singular antecedent given certain discourse and semantic properties (1b).


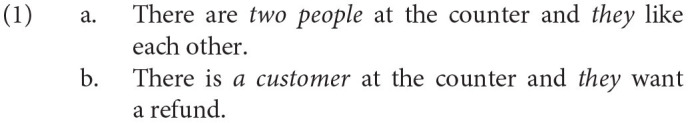


Known as “singular *they*”, this second usage is undergoing changes in progress in the types of linguistic antecedents it takes and the individuals it references (Camilliere et al., 2019; Conrod, 2019; Konnelly and Cowper, 2020). It is also the pronoun of reference for many individuals.

There is some evidence that *they* is underspecified along several dimensions and that this confers processing advantages upon it. Moxey et al. (2004) found, in comparison to instances where *they* lacked a salient plural antecedent, earlier disruptions in reading instances of *she/he* lacking a salient singular antecedent. Similarly, using ERP methods, Filik et al. (2008) found evidence of a cost for unheralded *she/he* but not for *they*. Sanford et al. (2008) looked at so-called “institutional *they*”, where the reference to an individual or group is highly underspecified (as in *On the train, they served coffee*), and found no processing costs compared to unheralded singular pronouns. These authors suggest that *they* is an underspecified pronoun and so will tolerate a wide range of antecedent types, posing no immediate processing difficulty [perhaps under shallow or good-enough processing (Ferreira et al., 2002)], yet possibly requiring greater resources in later processing (Moxey et al., 2004). Other such underspecified pronouns have been attested by Poesio et al. (2006) and the time course of their integration into sentence comprehension suggests similar conclusions.

At the same time, there is also evidence that in other contexts *they* exhibits a preference for plural antecedents. In an eye-tracking while reading study, Sanford and Filik (2007) found evidence of processing difficulty for *they* when only a singular antecedent was available. They argue that *they* expects a plural antecedent but can later accommodate a singular antecedent when no plural is found. In an ERP study, Chen et al. (2021) elicited a P600 effect for *they* with a singular antecedent in comparison to a plural antecedent, suggesting some processing difficulty for singular *they*. Similarly, in a Maze task study, Van Handel et al. (2021) report results that suggest that reflexive *themselves* preferentially links to a plural antecedent over a competing singular antecedent.

The gender of the antecedent also plays a role in the processing of *they* and *themselves* pronouns^1^. Prasad et al. (2018) found that the reflexive *themselves* elicited a P600, often taken to arise from syntactic anomaly, with singular antecedents that are associated with a gender stereotype (*John*) but not with non-gendered antecedents (*the participant*). They argue that *they/themselves* is underspecified for gender, and the processing cost is gender rather than number mismatch.

The studies cited above are limited to instances of “referential” *they*. As documented by Conrod (2019); Camilliere et al. (2019), and Konnelly and Cowper (2020), referential singular *they* is both undergoing many changes in present day English and is subject to several interacting discourse, pragmatic and sociolinguistic factors (Bjorkman, 2017; Camilliere et al., 2019; Conrod, 2019; Konnelly and Cowper, 2020). We focus on bound variable singular *they*, which has been attested for centuries (Balhorn, 2004; Bjorkman, 2017) and is stably acceptable among English users (Conrod, 2019; Konnelly and Cowper, 2020). Bound variable pronouns are unlike referential pronouns in that their denotation is not fixed. While *it* (*its* in possessive form) in (2a) picks out the one dog introduced by the antecedent noun phrase, the meaning of *it* in (2b) varies, picking out each of the individual dogs described by the antecedent noun phrase.


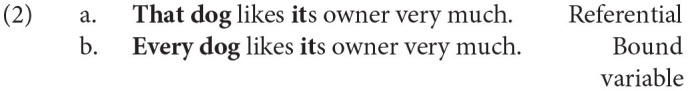


In the linguistics literature, the use of *it* in (2b) is described as a “bound variable” pronoun. This is appreciated by formulae such as *For every dog x, x likes* ***x***'*s owner*, where the third variable *x* stands for *it* in (2b). The pronoun in this case is said to be “bound” by the quantified phrase *every dog*.

Singular *they* can likewise be understood either as referential (3a) or as a bound variable (3b).


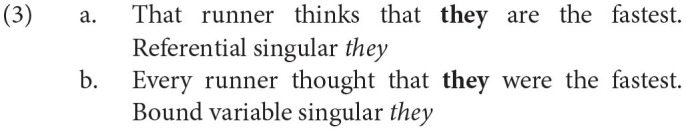


That *they* is interpreted as singular in both sentences in (3) is illustrated by the plausibility of the thought attributed to each runner: that they themselves, not a plurality of runners, are the unique fastest runner. Previous studies (Camilliere et al., 2019; Conrod, 2019; Han and Moulton, in press) have shown that English users generally prefer *they* over alternatives such as *he* or *she* as bound variables, particularly when the antecedent phrase does not express gender, as with *runner* in (3b). The processing studies on bound singular *they* suggest that it also has an advantage over referential *they*, at least for the English speakers who participated in those studies. For instance, Foertsch and Gernsbacher (1997) found that *they* can resolve to singular quantified antecedents without apparent difficulty regardless of the gender of the antecedent. Ackerman (2018) compared sentences employing *themself* with gendered and non-gendered antecedents, finding a processing advantage using eye-tracking while reading for gender stereotyped quantified antecedents (indefinites like *a mechanic*) as compared to referential antecedents (stereotyped proper names).

What has not been asked yet is whether there is any evidence that the number properties of the antecedent in quantificational sentences have an effect on the acceptability and processing of bound variable *they*, as has been suggested in the case of referential singular *they*. There is some suggestive evidence that they do. Using reading times and acceptability ratings, Han and Moulton (in press) compared singular *they* and singular gendered pronouns in sentences containing both gendered and non-gendered quantified (4a) and referential antecedents (4b).


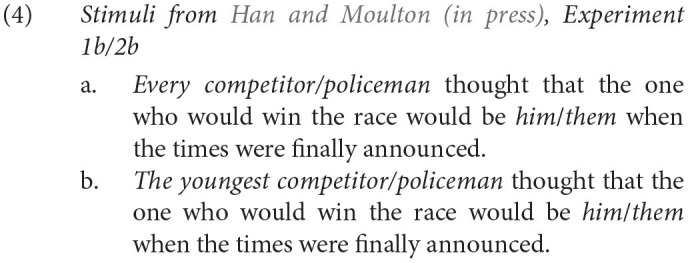


In reading time measures, Han and Moulton (in press) did not find any evidence of processing difficulty for bound singular *they* regardless of the gender of the quantified antecedent in the target region containing the critical pronoun, while they did find evidence of processing difficulty for referential singular *they* with gendered antecedents. However, in the spillover region, there was a main effect in which the *they*-sentences were read more slowly across the board, in comparison to the *he*-sentences. This was in contrast to the acceptability ratings that showed that bound singular *they* was rated as high as or even higher than *he*, while referential singular *they* was rated lower than *he*. Han and Moulton (in press) make the tentative suggestion that there may be a weak cost in initial processing when bound *they/them* finds only a singular antecedent.

No study reported to date, however, compares bound variable singular *they* with a plural vs. singular antecedent, and so cannot directly answer the question of whether bound variable singular *they* incurs a cost when taking a morphologically singular quantified antecedent like *every*. In the studies that follow, we make this comparison by capitalizing on configurations in English where a morphologically plural quantified noun phrase, headed by *all*, can bind *they* with a singular interpretation. This is shown in (5a) (Rullmann, 2003; Sudo, 2014). As with the bound variable sentence in (3b), repeated in (5b), this sentence attributes a pragmatically plausible thought to all the runners—that only one singular individual, they themselves, is the fastest runner.


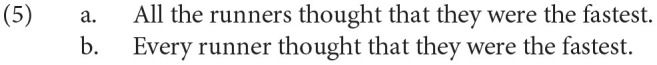


Sentences such as (5a) provide a near minimal contrast with (5b), allowing us to hold constant bound *they* but vary the number on the quantified antecedent. All else being equal, if *they* more readily accepts a plural antecedent at least initially—as has been hypothesized for referential singular *they* by Sanford and Filik (2007) and Van Handel et al. (2021)—we should expect an advantage for singular *they* bound by *all* as compared to *every*.

While contrasting these two quantified expressions offers a novel way to examine the role of morphological number in processing bound variable *they*, the two quantifiers differ along a semantic dimension, namely the difference between collective and distributive interpretations. *Every* often resists a collective reading, while *all* generally allows one. With a predicate such as *swarm*, which requires a plural argument, a noun phrase quantified by *every* is not acceptable, whereas one quantified by *all* is (6) (Morgan, 1984; Winter, 2002).


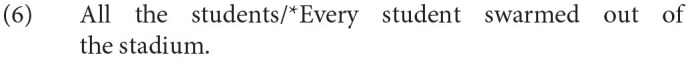


The more distributive nature of *every* could make singular interpretations of *they* more available in comparison to *all*, on the assumption that it promotes an interpretation in which the predicate that corresponds to the nuclear scope of the quantifier holds of singular (atomic) individuals. This could have the effect of elevating the acceptability of a binding dependency between *every* and singularly-interpreted *they* while depressing that between *all* and *they*. As a consequence, this might obfuscate an advantage in number congruency between *all* and *they*^2^. Support for this concern can be found in the existing experimental literature. Patson and Warren (2010) test the interpretation of indefinite expressions such as *a box* when presented as the direct object of a sentence with either a distributive (with *each*) or a collective (with *together*) subject. Their study shows that participants are more likely to interpret the indefinite singular as referring to multiple different objects mapped to covarying antecedents under the distributive subject vs. the collective one. This is direct evidence of a distributive subject invoking multiple individuals, which could each in turn serve as an ideal referent for singular *they* in a binding context, as noted above. In short, the salience of the multiple atomic individuals invoked by a quantifier like *every* or *each* may be promoting a preference for a bound singular reading to a degree that outmatches a preference for a plural reading of *they* when anteceded by a subject with *all*.

In light of these considerations, one goal of our studies was to investigate the role of distributivity in the availability of bound singular *they*. To do so, we additionally tested quantified phrases headed by *each*, which shows very strong tendencies toward distributivity (Vendler, 1967; Ioup, 1975; Tunstall, 1998).^3^ We reason that if distributivity makes singular bound variable *they* more available, we should find a cline in acceptability that mirrors the cline in distributivity from *all* to *every* to *each*. That is, in terms of acceptability judgments, we predict that singularly-interpreted *they* bound by *each* will be rated as more acceptable than that bound by *every*, which will be in turn rated as more acceptable than singular *they* bound by *all*. On the other hand, if congruence in superficial morphological number alone regulates the acceptability of bound variable *they*, then we predict sentences where *all* binds singularly-interpreted *they* will be rated higher than those involving *each* and *every*.

As for predictions concerning the incremental processing of sentences involving bound *they* under these different quantifiers, we contrast the predictions of the view that, for at least some speakers, *they* initially seeks plural antecedents and resists singular ones (Sanford and Filik, 2007; Chen et al., 2021; Van Handel et al., 2021) with those of an underspecification approach. Under the former view, we expect to find longer reading times for the pronoun when only a singular antecedent is available. (We use morphologically singular pronouns like *he* to test for a baseline reading time penalty for pronouns that can find only a number mismatched plural antecedent.) On the other hand, under the underspecification account, if at the earliest moments of processing, *they* is underspecified for number as suggested by Moxey et al. (2004) and Sanford et al. (2008), we do not expect any disruption in reading times at the pronoun in sentences where it can only find an antecedent headed by singular *every/each* in comparison to plural *all*. Under either account, however, if distributivity is a factor in incremental processing, then we expect some processing penalty for *all* in comparison to *every/each*.

We first report two acceptability rating studies in which we determine the acceptability of bound variable singular *they* under three types of quantifiers: plural collective (*all*), singular collective/distributive (*every*), and singular distributive (*each*). Crucially, we presented participants with sentences that forced a singular interpretation of *they*. One study tested sentences with non-gendered quantified antecedents and the other tested sentences with gendered quantified antecedents. In both studies, we observed a cline in acceptability of bound variable singular *they* such that *each* is better than *every*, while *every* is variably better than *all*. While distributivity does affect the acceptability of *they*, bound *they* under *all* is relatively acceptable, particularly in comparison to a non-quantified baseline. As for morphological number, we found no evidence of reduced acceptability for *they* taking a singular quantified antecedent.

We then turn to two self-paced reading studies to investigate whether there are any processing costs associated with *they* taking a singular as compared to a plural quantified antecedent. The two studies tested sentences with non-gendered antecedents and sentences with gendered antecedents, respectively. While the acceptability judgments suggest no such difference, incremental processing at the pronoun may be delayed if the pronoun's morphological number is not congruent with the only available antecedent, a cost that is potentially overcome by the time readers make considered acceptability judgments. Furthermore, given our acceptability judgments, distributivity could also be reflected in processing costs. As we report below, in the two self-paced reading studies, we found no evidence of a difference between plural and singular quantified antecedents for *they*. Similarly, we found no processing costs reflecting the distributive/collective distinction. Rather, for all types of quantified antecedents, encountering a singular pronoun (*he*) rather than *they* registered a processing delay, in contrast to non-quantified antecedents.

We suggest that the absence of number effects in both the online and offline measures is most compatible with the view that *they* is underspecified for number, and the presence of distributivity effects only in the acceptability ratings is related to a perhaps late construal mechanism that is required to fully semantically interpret singular-denoting pronouns under non-distributive quantifiers like *all* (Rullmann, 2003; Sudo, 2014). Furthermore, we take the processing effect associated with bound singular pronouns (*he*) to reflect a general fact about the English pronoun system, revealed here at the early moments of processing: morphologically singular pronouns resist bound interpretations, possibly because *they* is the language's preferred alternative.

## 2. Experiment 1: Rating, Non-gendered Antecedent

Experiment 1 tested the acceptability of bound singular *they* in the context of non-gendered antecedent quantifier phrases headed by *all, every*, and *each*. As a baseline, we also tested the acceptability of singular *they* in sentences with definite plural antecedents. We predict that if bound singular *they* prefers a plural antecedent, *all* should be more acceptable than *every* and *each*. By contrast, if bound singular *they* prefers a distributive antecedent, *each* should be the most acceptable and *all* the least acceptable of the quantifiers under investigation. As we detail below, our stimuli were created in a very specific way to help ensure that readers interpreted *they* as a singular bound variable.

### 2.1. Methods

#### 2.1.1. Participants

Forty-eight native English speakers (27 women, 19 men, 1 non-binary, 1 unclassified)^4^ were recruited through Prolific and completed the experiment on PCIbex Farm (Zehr and Schwarz, 2018). Participants range in age from 19 to 54 years, with a mean age of 32.06 years. Participants were compensated £2.50 upon confirmation of experiment completion.

#### 2.1.2. Task, Design, and Materials

Experiment 1 involved a sentence acceptability judgment task with a total of 80 items: 24 test items and 56 fillers. The 24 test item sets were constructed with a single factor, Antecedent, with four levels depending on the form of the antecedent noun phrase: def.plural (plural definite descriptions); and three types of quantified phrases: all with a plural noun and every and each with singular nouns. Each item consisted of a context sentence and a target sentence. The context sentence introduced an indefinite group described using a non-gendered common noun (e.g., *a big group of cyclists*). Non-gendered common nouns (e.g., *cyclist*) readily license singular *they* (Doherty and Conklin, 2017). The target sentence reiterated the non-gendered noun in a plural definite description (*the*, def.plural) or a quantified phrase (all, every, each). A sample test item set is given in (7). To force a singular interpretation for *they*, we predicated it of the expression *be the only one* in the embedded clause. (Note the infelicity of #*The cyclists are the only one who like the pouring rain*).


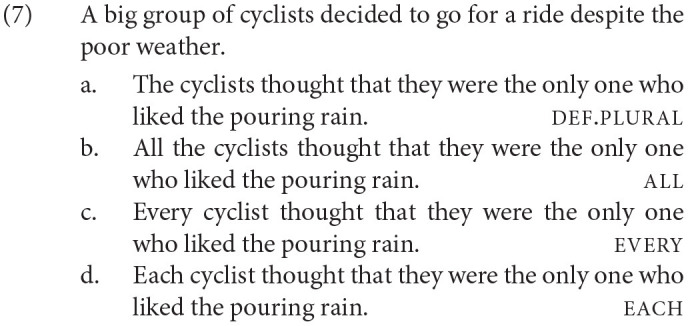


The def.plural condition was intended to serve as an ungrammatical baseline, on the assumption that a referential, plural-denoting *they* would be most naturally anaphoric to such an antecedent. Such a referent would then not be compatible with the predicate *be the only one*.

An additional 56 fillers included 10 natural items (8) as well as ten unnatural items, evenly divided among garden path (9) and otherwise unnatural sentences (10). The remainder of the fillers were drawn from a separate experiment. None of the fillers involved singular *they*.


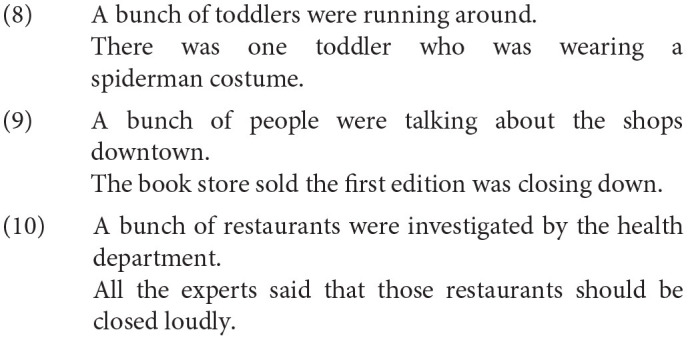


#### 2.1.3. Procedure

The test items for Experiment 1 were distributed across four lists in a Latin-Square design. Each list contained 24 test items and 56 fillers, which were presented to the participant in a randomized order.

Experiment 1 was implemented as an online experiment on PCIbex Farm (Zehr and Schwarz, 2018). Each trial presented the participant with the context sentence and the target sentence below it. The target sentence was presented in bold-face while the context sentence was not. Participants were instructed to read through the sentence pair and rate the naturalness of the target sentence on a seven-point scale, with 7 being the most natural and 1 the most unnatural. Seven practice items were presented before the beginning of the experiment to familiarize participants with the task. After the experiment, participants were asked to complete a demographic survey concerning their age, language background, and gender.

### 2.2. Results

**Figure 1:**
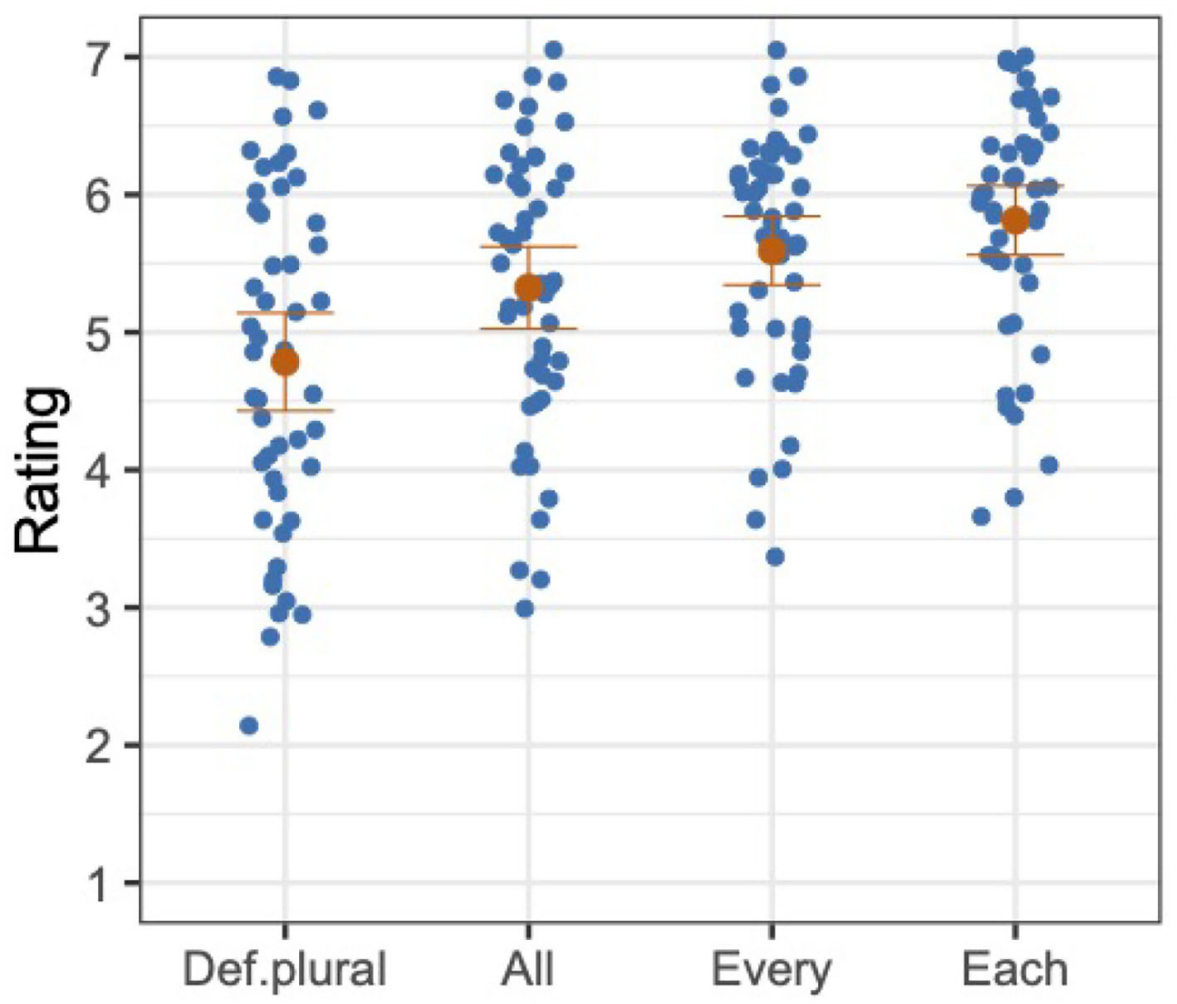
Distributions of mean ratings of participants (in blue), and mean ratings across participants with standard errors (in orange), Experiment 1.

The overall mean and the distribution of participants' mean ratings are provided for each condition in Figure 1. Each blue dot represents a mean rating of a participant in a given condition, and each orange dot represents the mean rating across participants in a given condition.

We analyzed the z-transformed ratings using a mixed-effects model in R (R Development Core Team, 2020). The lme4 package was used to fit the model (Bates, 2005), and the lmerTest package was used to obtain *p*-values (Kuznetsova et al., 2014). In analyses of data obtained from all experiments reported in this article, we fit a maximal random-effects structure with random intercepts and random slopes for participants and items (Barr et al., 2013).

The model was fit to the z-transformed ratings with the fixed factor of Antecedent. This predictor was forward-difference coded, such that def.plural was compared to all (Antecedent1), all was compared to every (Antecedent2), and every was compared to each (Antecedent3).^5^ We found a significant or marginal difference for all comparisons, as shown in Table 1. The mean z-transformed rating of def.plural was significantly lower than the mean z-transformed rating of all, the mean z-transformed rating of all was marginally lower than the mean z-transformed rating of every, and the mean z-transformed rating of every was significantly lower than the mean z-transformed rating of each.

**Table 1:** Fixed effects, Experiment 1.

	**Estimate**	**Std. Error**	**df**	***t*-value**	**Pr(>|t|)**
(Intercept)	0.47377	0.04214	53.22669	11.242	1.13e-15^***^
Antecedent1	–0.24489	0.07506	36.81260	–3.263	0.00238^**^
Antecedent2	–0.13721	0.06809	36.12135	–2.015	0.05139^+^
Antecedent3	–0.11586	0.05212	52.36299	–2.223	0.03057^*^
Formula in R: Rating ~ Antecedent + (1+Antecedent|Participant) + (1+Antecedent|Item)					

*Significance levels*:

*** *p < 0.001*,

** *p < 0.01*,

* *p < 0.05*,

+ *p < 0.1*.

### 2.3. Discussion

The acceptability ratings of considered judgments from our participants show that bound singular *they* is generally acceptable with non-gendered quantified antecedents, with *each* being the most acceptable and *all* the least acceptable. This pattern of results shows that bound singular *they* does not prefer a plural quantifier, but is more acceptable with quantifiers of greater distributivity. The validity of these results is supported by the filler sentence ratings patterning as expected. While the garden-path and unnatural fillers were rated low, 2.71 and 3.62, respectively, the natural fillers were rated high, 6.09.

The definite plural condition, while rated lower than all other conditions, was nonetheless relatively acceptable, particularly compared to the unnatural fillers. We speculate that definite plural phrases can be construed distributively, perhaps *via* a silent distributive operator (DIST) that is proposed extensively in the semantics literature (Roberts, 1987; Rullmann, 2003, among others), as in (11).


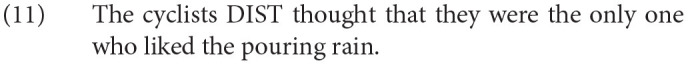


It has been shown that plurals have a default preference for collective interpretations (Frazier et al., 1999; Dotlačil and Brasoveanu, 2021), thus a distributive operator would be required in the semantics to make these definite plurals appropriate antecedents for a bound singular *they*, as reinforced by the post-copular predicate. Accommodating a distributive operator in the context of a definite plural could be something that readers perform with effort. A need for such accommodation for sentences that require singular bound variable interpretations based on material coming after the processing of both the antecedent and the pronoun could then lead to reduced acceptability^6^.

## 3. Experiment 2: Rating, Gendered Antecedent

Experiment 2 examined the naturalness of singular *they* with gendered antecedents. Previous studies show that the gender of the antecedent can affect the acceptability of *they* (Doherty and Conklin, 2017; Ackerman, 2018; Ackerman et al., 2018; Prasad et al., 2018). At the same time, Han and Moulton (in press) found that the gender of the antecedent had no effect on the acceptability and processing of singular *they* bound by the quantifier *every*. We therefore set out to determine whether gender modulates the acceptability of singular *they* with the plural (non-distributive) quantifier *all*. This will help delimit the role of gender in regulating the acceptability of bound singular *they*. We expect that when *they* is bound by *all*, then sentences with gendered common nouns should exhibit the same pattern as the ones with non-gendered common nouns as found for *every* in Han and Moulton (in press).

### 3.1. Methods

#### 3.1.1. Participants

Forty-eight native English speakers (29 women, 17 men, 1 non-binary, and 1 unclassified) were recruited through Prolific^7^ and completed the experiment on PCIbex Farm (Zehr and Schwarz, 2018). Participants range in age from 18 to 66 years, with a mean age of 32.50 years. Participants were compensated £2.50 upon confirmation of experiment completion.

#### 3.1.2. Task, Design, and Materials

Experiment 2 involved a sentence acceptability judgment task similar to Experiment 1 with a total of 80 items: 24 test items and 56 fillers.

The 24 test item sets were constructed like in Experiment 1 with a single factor and four levels: Antecedent (def.plural, all, every, or each). Each item consisted of a context and a target sentence, both of which were structured the same as those in Experiment 1. In Experiment 2, however, the common nouns introduced by the context sentence and then reiterated in the target sentence were gender stereotypical male (e.g., *a group of workmen*). A sample test item set is given in (12).


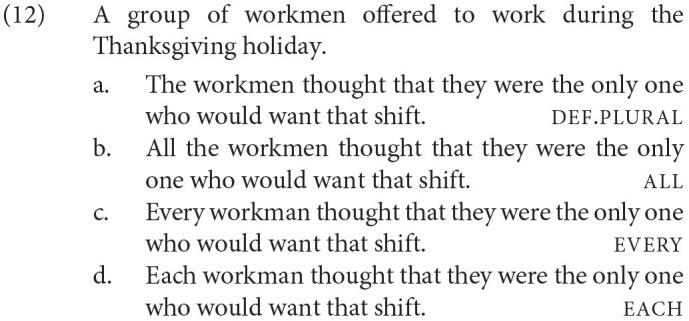


Fillers were the same as those used in Experiment 1. Again, no fillers involved the use of singular *they*.

#### 3.1.3. Procedure

Experiment 2 followed the same procedure as Experiment 1.

### 3.2. Results

**Figure 2:**
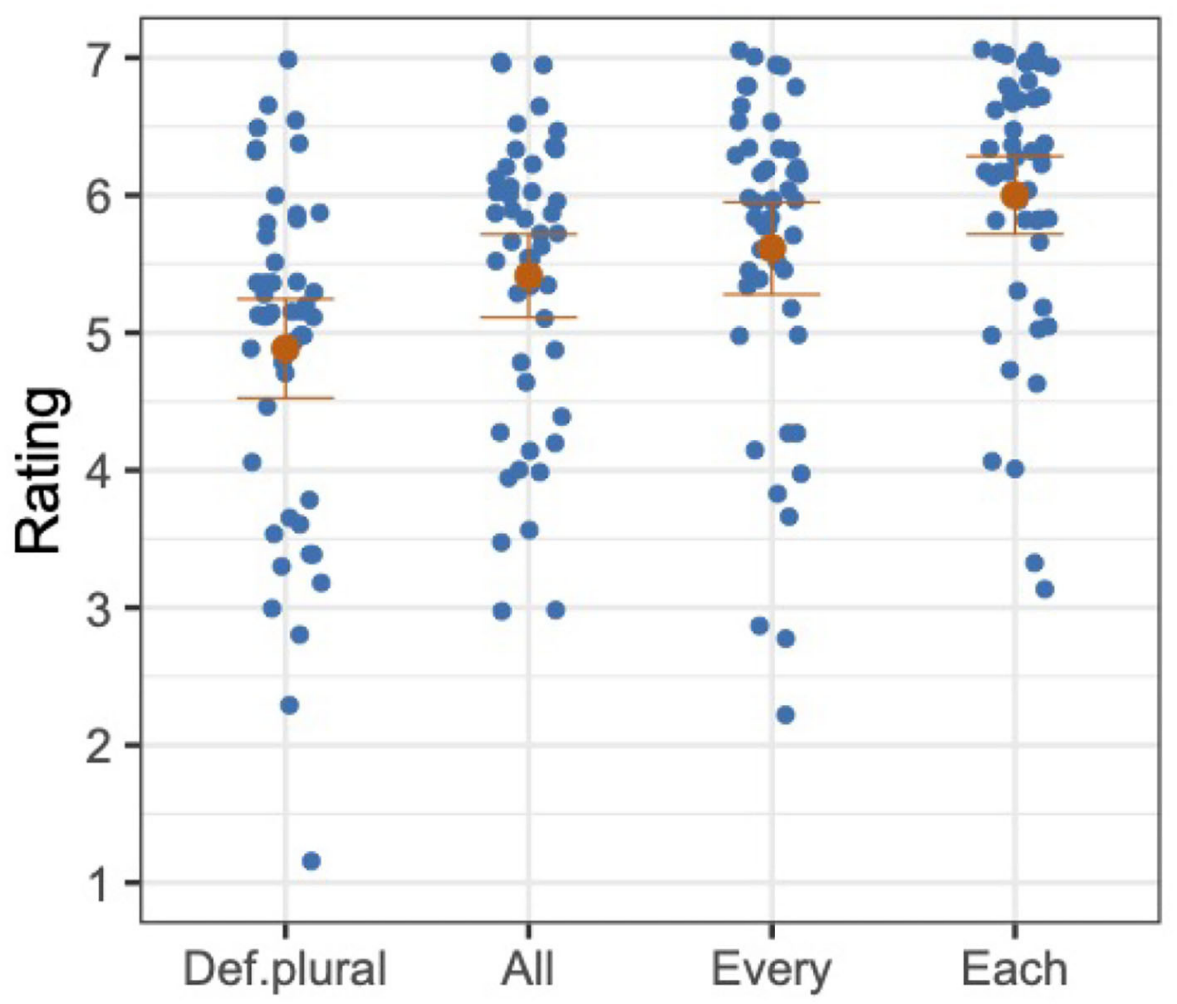
Distributions of mean ratings of participants (in blue), and mean ratings across participants with standard errors (in orange), Experiment 2.

The overall mean and the distribution of participants' mean ratings are provided for each condition in Figure 2. Each blue dot represents a mean rating of a participant in a given condition, and each orange dot represents the mean rating across participants in a given condition.

We analyzed the z-transformed ratings with a mixed model, with a random-effects structure as described for Experiment 1. The model was fit to the z-transformed ratings with the fixed factor of Antecedent, which was forward-difference coded in the same way as in Experiment 1. As shown in Table 2, we found a significant difference in the comparison between def.plural and all (Antecedent1) and for the comparison between every and each (Antecedent3) but not for the comparison between all and every (Antecedent2). That is, the mean z-transformed rating of def.plural was significantly lower than the mean z-transformed rating of all, and the mean z-transformed rating of every was significantly lower than the mean z-transformed rating of each. However, even though the mean rating of every was numerically higher than the mean rating of all, the difference between the mean z-transformed ratings of the two conditions was not statistically significant.

**Table 2:** Fixed effects, Experiment 2.

	**Estimate**	**Std. Error**	**df**	***t*-value**	**Pr(>|t|)**
(Intercept)	0.47351	0.03611	46.84619	13.112	<2e-16^***^
Antecedent1	–0.25782	0.06199	35.20638	–4.159	0.000195^***^
Antecedent2	–0.08027	0.05946	38.66600	–1.350	0.184851
Antecedent3	–0.19324	0.05203	54.39477	–3.714	0.000483^***^
Formula in R: Rating ~ Antecedent + (1+Antecedent|Participant) + (1+Antecedent|Item)					

*Significance levels*:

*** *p < 0.001*,

**p < 0.01, *p < 0.05, ^+^p < 0.1.

### 3.3. Discussion

The results of Experiment 2 are similar to the results of Experiment 1. Just as with non-gendered quantified antecedents, we found that bound singular *they* with gendered quantified antecedents is generally acceptable, with *each* being the most acceptable and *all* the least acceptable. Although the difference between *all* and *every* was not statistically significant, we see that the mean rating of *every* is numerically higher than the mean rating of *all*. The overall pattern of results in Experiment 2, thus, shows that bound singular *they* does not prefer a plural quantifier, and is more acceptable with quantifiers of greater distributivity. This leads us to conclude that the gender of the antecedent is not a critical factor in the acceptability of bound singular *they*; the pattern of acceptability of the sentences with gendered nouns is similar to the ones with non-gendered nouns^8^. Moreover, just as in Experiment 1, the definite plural condition had the lowest mean rating, but still was rated to be relatively acceptable. Here again, we speculate that a distributive operator can be accommodated to construe definite plural phrases distributively, but this need for accommodation may have contributed to a reduction in acceptability^9^.

The results for the fillers in Experiment 2 are also similar to the ones in Experiment 1: the natural fillers were rated high, 6.21, and the unnatural and garden-path fillers were rated low, 3.58 and 2.84 respectively.

Having shown that in an offline acceptability rating task, bound variable *they* is not sensitive to the number of an antecedent quantifier, we turn to self-paced reading studies to determine whether there is any initial effect of number during online processing that is not captured in a rating task. Furthermore, these studies probe for an online processing effect of distributivity, reasoning based on the results of Experiments 1 and 2 that late revelation of the need to interpret an antecedent distributively rather than collectively might incur a processing cost, as argued in Dotlačil and Brasoveanu (2021). The quantifier *each* provides an early signal of distributivity since this is part of that quantifier's lexical meaning. The processor does not need to accommodate distributivity downstream. The quantifier *all*, on the other hand, does not lexically encode distributivity. A distributive semantics, to the extent it is possible with *all*, arises *via* inferences about the sentence more globally. It is natural to expect that this requires some sort of reanalysis out of which we expect processing disruption.

## 4. Experiment 3: Self-paced Reading, Non-gendered Antecedent

Experiment 3 investigated the incremental processing of *they* in comparison to a superficially singular pronoun (*he*) in sentences with non-gendered quantified antecedents. Using a self-paced reading task, we examined the processing profile of *they* in the context of three types of quantified antecedents (*all, every*, and *each*), with the goal of addressing whether bound singular *they* incurs a processing cost with singular as compared to plural quantified antecedents, and additionally, whether the semantic distributivity of the antecedent quantifier has any effect. We intended the singular pronoun (*he*) to serve as a control because we expected it to exhibit processing difficulty in the context of *all* but not in the context of *every* or *each*.

Here, we follow studies like those in Foertsch and Gernsbacher (1997) and Sanford and Filik (2007) where we measure the reading time of pronouns that are given only one antecedent. Similarly, we will interpret longer reading times at the pronoun as showing that the processor has more difficulty integrating—or linking—the pronoun to the one available antecedent. Such a difficulty is expected if *they* initially accepts only a plural antecedent, and induces a cost when only a singular antecedent is available (Sanford and Filik, 2007). In that case, we expect slower reading times at *they* (or soon after) following *each/every* as compared to *all*. If *they* is underspecified for number in initial processing, we do not expect an impact of number in the form of longer reading times at the point of the pronoun.

We test these predictions using the stimuli from the acceptability rating study Experiment 1. As in the acceptability rating studies, we included material in our self-paced reading stimuli to enforce a bound singular interpretation of *they*. Recall that in those stimuli a bound singular interpretation is essentially forced by the predicate *be the only one*. While this does not force a bound interpretation in advance of encountering the pronoun (since it follows the pronoun), we sought to use this material as a probe to determine if readers made an early commitment to a plural interpretation of *they*. If readers simply pursued a plural interpretation of *they*—referentially anaphoric to the plurality introduced by, e.g., the *all* phrase—they should demonstrate difficulty at *only one*.

### 4.1. Methods

#### 4.1.1. Participants

A total of 121 native English speakers (51 women, 68 men, 1 transmasculine, and 1 unclassified) were recruited through Prolific and completed the experiment on Ibex Farm (Drummond, 2013). Participants range in age from 18 to 72 years, with a mean age of 35.77 years. Participants were compensated £2.50 upon confirmation of experiment completion.

#### 4.1.2. Task, Design, and Materials

Experiment 3 involved a self-paced reading task with a total of 72 items: 36 test items and 36 fillers. The 36 test item sets were constructed with a 2 × 3 factorial design of Antecedent (all, every, and each) and Pronoun (they, he). Each item consisted of a context sentence with one region and a target sentence with ten regions. The context sentences were identical to the ones in Experiment 1, introducing an indefinite group containing a non-gendered common noun. The target sentence reiterated the non-gendered noun as part of a quantifier phrase in Region 1, which antecedes the pronoun in Region 3. Region 7 contains the words *only one* across all conditions to enforce a singular bound interpretation of the pronoun in Region 3, identical to Experiment 1. Region 3 and Region 7 are the two regions of interest. We will refer to Region 3 as the “pronoun region” and Region 7 as the “singular region” (since it disambiguates *they* to a singular interpretation). A sample test item set is given in (13).


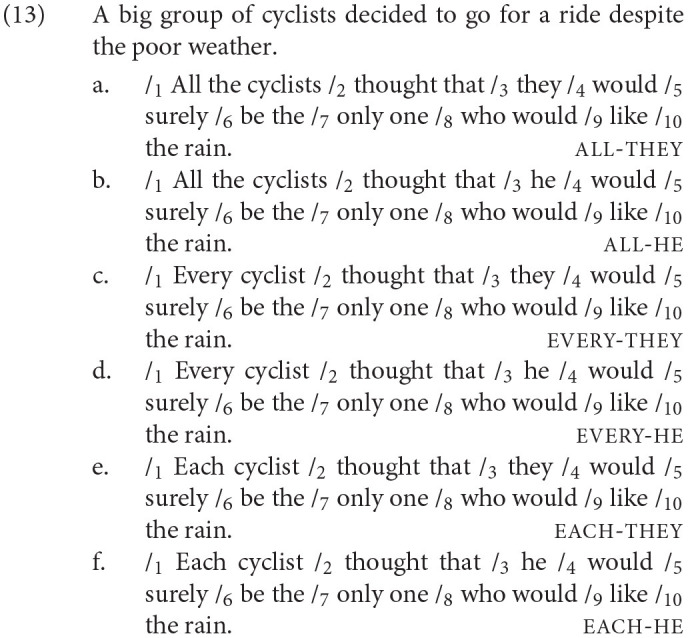


A total of 36 sentences from a separate experiment on resumptive pronouns were included as fillers.

#### 4.1.3. Procedure

The 36 test items were distributed across six lists in a Latin-Square design, with an additional 36 fillers. Each list was randomized and had two variations, presented either in forward or reverse order.

Participants completed the experiment *via* their internet browsers through Ibex Farm (Drummond, 2013). Each trial presented the participant with a context sentence followed by the target sentence in a region-by-region self-paced reading paradigm. When a participant pressed the space bar, one region of words would be presented on the screen. Upon the next space bar press, this region would disappear, and the subsequent region would be shown. Once the participant had viewed all the regions, a *yes-no* comprehension question was presented, asking about the content of the context sentence and not the interpretation of the critical pronoun. The corresponding comprehension question to the item in (13) is provided in (14).


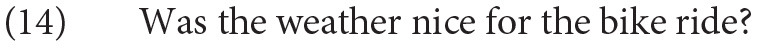


Two practice items were presented at the beginning of the experiment to familiarize participants with the task. After the experiment, participants were instructed to complete a demographic survey identical to the one presented in Experiment 1. Participants were also required to confirm their Prolific ID to receive compensation.

### 4.2. Predictions

The predictions for the pronoun region are as follows. If upon first encountering *they*, readers initially expect it to form a dependency with a plural antecedent, then in the they condition, the *every*- and *each*-sentences should exhibit processing costs in comparison to the *all*-sentences in the pronoun region and/or its spillover region. By contrast, if *they* is underspecified in number, then the *every*- and *each*-sentences should be processed as easily as the *all*-sentences in the they condition. Regardless of whether bound singular *they* exhibits number mismatch effects with singular quantifiers, in the he condition, the *all*-sentences should incur processing costs in comparison to the *every*- and *each*-sentences in the pronoun region and/or its spillover region, due to the number mismatch between plural *all* and singular *he*.

As for the predictions for the singular region, if *they* is interpreted as a singular bound variable, no processing difficulty should be attested in the singular region and its spillover region. However, if *they* is interpreted as a referential plural pronoun, then we should detect a processing difficulty in the singular region and/or its spillover region, as this would result in a clash between a plural pronoun and *be the only one*. Building upon the acceptability judgment studies (Experiments 1 and 2), we reason that the less distributive the antecedent quantifier is, the more likely that *they* is interpreted as a plural referential pronoun, leading to more processing difficulty. In Experiments 1 and 2, we found a cline in acceptability of bound variable *they* that mirrors the cline in distributivity from *all* to *every* to *each*. Given this finding, we expect the *each*-sentences to be the easiest to process followed by the *every*-sentences, and then the *all*-sentences in the they condition. On the other hand, the he condition should exhibit no processing difficulty regardless of whether *he* is interpreted as a bound pronoun or a referential pronoun, as singular *he* is semantically compatible with *be the only one*. As such, we do not expect to see any differences in the processing profile of *he* among the tested antecedent quantifiers.

### 4.3. Results

Five participants whose range of mean reading times across regions was less than 50 ms were eliminated from the analysis. In addition, using the trimr package (Grange, 2015), reading times of a region that were 10 SDs above the mean were removed, in order to exclude extreme outliers from the analysis. This resulted in further removing 0.1% of the observations from the data.

The grand mean comprehension question response score on test sentences was 89%. The mean proportions of correct responses for the comprehension questions are reported in Table 3. The comprehension questions tested participants' attention to the overall sentence content, and the results show no impact of the manipulated factors on comprehension.

**Table 3:** The proportion of correct responses (SE), Experiment 3.

	**HE**	**THEY**
all	0.89 (0.004)	0.89 (0.004)
every	0.89 (0.004)	0.91 (0.004)
each	0.89 (0.004)	0.90 (0.004)

**Figure 3:**
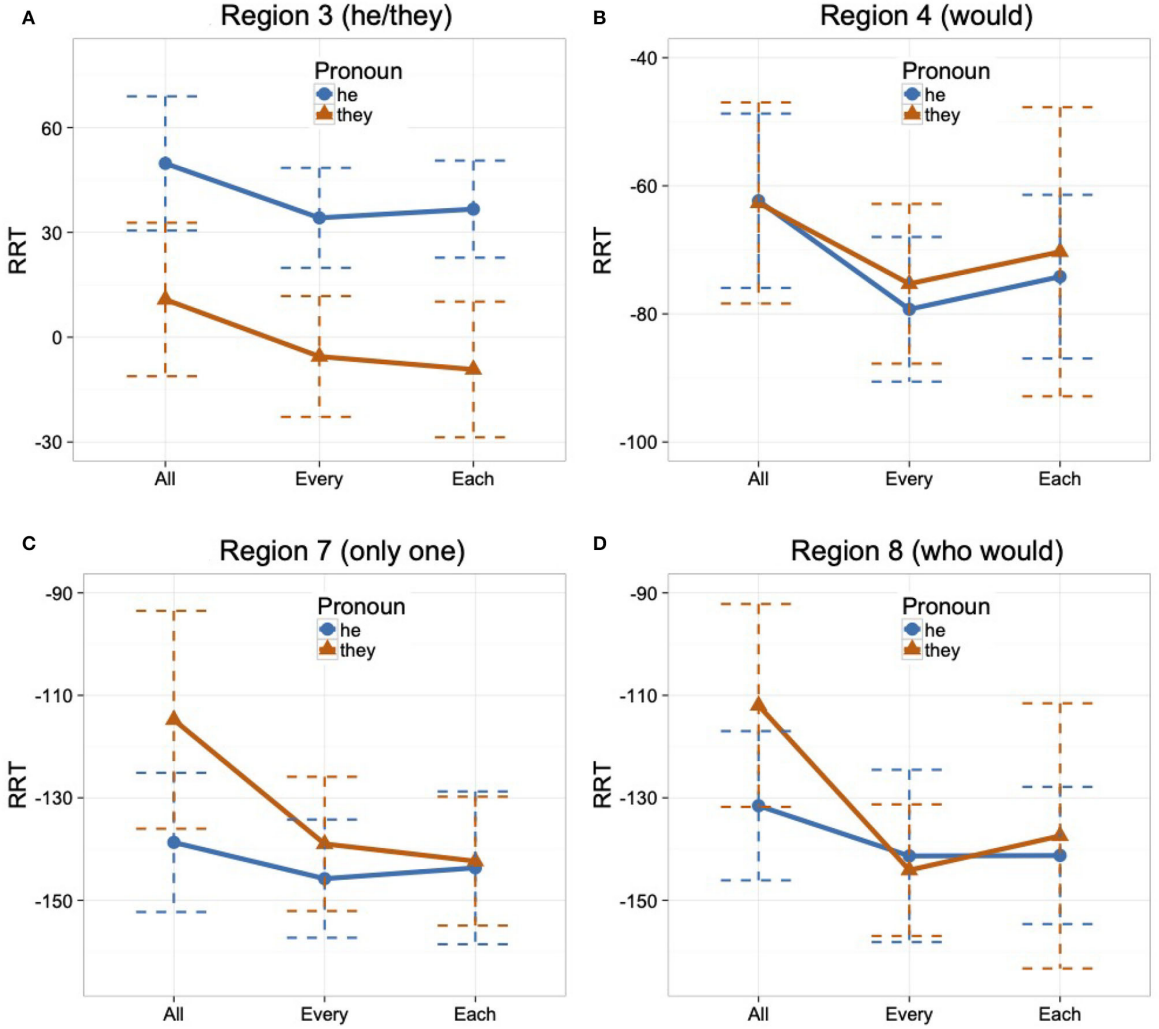
Mean RRTs by the condition in Regions 3, 4, 7, and 8 with standard errors **(A–D)**, Experiment 3.

Mean raw reading times by condition for each region (excluding the first and the last region) are reported in Table 4. These represent reading times for all data, regardless of whether the comprehension question was answered correctly. We calculated residual reading times (RRTs) using character-length from the entire dataset (including fillers) to estimate the reading time for each region for each participant (Ferreira and Clifton, 1986; Trueswell and Tanenhaus, 1994; Phillips, 2006). The graph in Figure 3 summarizes mean RRTs by condition for the first region of interest (Region 3), which is the pronoun region, and its spillover region (Region 4), as well as the second region of interest (Region 7), which is the singular region, and its spillover region (Region 8).

**Table 4:** Mean raw reading times (SE) in ms, Experiment 3.

**Region**		**2**	**3**	**4**	**5**	**6**	**7**	**8**	**9**
all	they	481 (17)	394 (12)	345 (9)	347 (8)	355 (10)	364 (12)	385 (11)	385 (8)
	he	462 (10)	386 (10)	345 (7)	347 (7)	348 (7)	340 (7)	366 (8)	375 (6)
every	they	468 (13)	378 (9)	332 (6)	334 (7)	349 (8)	340 (7)	353 (7)	374 (7)
	he	437 (8)	370 (7)	328 (5)	333 (7)	337 (7)	333 (6)	356 (9)	366 (5)
each	they	448 (14)	374 (11)	337 (12)	349 (11)	347 (13)	336 (6)	359 (14)	377 (9)
	he	517 (53)	373 (7)	333 (6)	339 (9)	349 (8)	335 (8)	356 (7)	373 (7)

We analyzed each region's RRTs with a mixed model, fitting a random-effects structure with random intercepts and random slopes for participants and items, with the random correlation parameter for the interaction term removed for both participants and items (Barr et al., 2013). The model was fit to the RRTs with the fixed factors of Antecedent and Pronoun. Antecedent was forward-difference coded, such that all was compared to every (Antecedent1), and every was compared to each (Antecedent2). Pronoun was sum coded, with the he level coded as 1, and the they level as –1.

In analyzing Region 3, the pronoun region, we found a main effect of Pronoun such that regardless of the antecedent type, the he condition showed a slower reading time than the they condition, as shown in Table 5^10^. In Region 4 (spillover region), the analysis showed no main effect or interaction. Region 7, the singular region, also showed no effect. Region 8 (spillover region) revealed a significant difference between the all condition and the every condition such that regardless of the pronoun type, the all condition showed a slower reading time than the every condition. But no difference was found between the every and the each condition.

**Table 5:** Fixed effects, Experiment 3, Regions 3, 4, 7, and 8.

**Region 3**	**Estimate**	**Std. Error**	**df**	***t*-value**	**Pr(>|t|)**
(Intercept)	19.3430	8.7276	114.8943	2.216	0.0286^*^
Antecedent1	15.8216	8.7563	139.4938	1.807	0.0729^+^
Antecedent2	0.8387	9.0083	89.3282	0.093	0.9260
Pronoun1	20.7587	4.0653	45.3817	5.106	6.33e-06^***^
Antecedent1:Pronoun1	–0.4215	8.4695	3902.4575	–0.050	0.9603
Antecedent2:Pronoun1	–3.1842	8.4680	3903.6940	–0.376	0.7069
**Region 4**	**Estimate**	**std. Error**	**df**	**t value**	**Pr(>** **|** **t** **|** **)**
(Intercept)	–70.7477	9.1028	117.6251	–7.772	3.22e-12^***^
Antecedent1	14.6934	7.4774	117.5584	1.965	0.0518^+^
Antecedent2	–4.9414	8.8771	68.3346	–0.557	0.5796
Pronoun1	–1.2451	3.9225	80.6040	–0.317	0.7517
Antecedent1:Pronoun1	2.1134	6.7826	3880.0219	0.312	0.7554
Antecedent2:Pronoun1	–0.1513	6.7843	3878.6941	–0.022	0.9822
**Region 7**	**Estimate**	**std. Error**	**df**	**t value**	**Pr(>** **|** **t** **|** **)**
(Intercept)	–137.595	9.010	124.496	–15.271	<2e-16^***^
Antecedent1	15.120	9.191	40.075	1.645	0.108
Antecedent2	1.131	6.959	283.437	0.163	0.871
Pronoun1	–5.324	3.272	66.087	–1.627	0.109
Antecedent1:Pronoun1	–8.560	6.704	3870.289	–1.277	0.202
Antecedent2:Pronoun1	–3.220	6.709	3868.270	–0.480	0.631
**Region 8**	**Estimate**	**std. Error**	**df**	**t value**	**Pr(>** **|** **t** **|** **)**
(Intercept)	–134.697	8.259	109.556	–16.309	<2e-16^***^
Antecedent1	20.957	9.313	154.627	2.250	0.0258^*^
Antecedent2	–3.154	10.721	69.314	–0.294	0.7695
Pronoun1	–3.423	4.707	72.587	–0.727	0.4694
Antecedent1:Pronoun1	–11.256	8.448	3873.329	–1.332	0.1828
Antecedent2:Pronoun1	3.128	8.447	3872.690	0.370	0.7112
Formula in R: RRT ~ Antecedent*Pronoun + (1+Antecedent+Pronoun|Participant) + (1+Antecedent+Pronoun|Item)					

*Significance levels*:

*** *p < 0.001*,

^**^p < 0.01,

* *p < 0.05*,

+ *p < 0.1*.

### 4.4. Discussion

In the pronoun region (Region 3), *they* with *every* or *each* as an antecedent quantifier was not read slower than *they* with *all*. We, thus, found no evidence that *they* gives rise to processing difficulty when only a singular antecedent is available, even when holding the quantificational nature of the antecedent constant.

Comparing *they* and *he* in the pronoun region, we found that *he* was read significantly slower than *they* with all of the tested antecedent quantifiers (*all, every*, and *each*). The processing difficulty incurred by *he* in the all condition is expected, as *all* is morphologically plural and so mismatches in number with *he*. However, the processing difficulty incurred by *he* in the every and each conditions cannot be attributed to a number mismatch with *he*, as both *every* and *each* are morphologically singular. Thus, the finding that *they* was read faster than *he* in the every and each condition suggests that *they* can easily retrieve singular quantifiers, more so than *he* can, and reinforces that *they* enjoys no advantage with a plural rather than singular quantified antecedent. Alternatively, the finding that *they* was read faster than *he* in the every and each condition might be attributed to a possible confound that under a non-gendered antecedent noun, integrating a masculine pronoun (*he*) is more difficult than integrating a non-gendered pronoun (*they*). We removed this possible confound in Experiment 4 by using test sentences containing male gendered antecedent nouns.

We used the singular region (Region 7) as a check to determine whether readers are pursuing a singular or plural interpretation of *they*. At the point where readers encounter *they* in the all condition, it could be interpreted as a co-referential plural pronoun, as *all* is the least distributive quantifier. We reasoned, however, that if participants did interpret it as such, they would have difficulty integrating the content of the singular region (*only one*). This would then lead to longer reading times in that region in the they condition compared to the singular pronoun condition. By the same reasoning, the *every*-sentences could incur some processing cost in comparison to the *each*-sentences, reflecting the collective/distributive distinction of these quantifiers. We found no evidence of difficulty for any of the tested quantifiers, as overall there was no statistical difference between the reading times of sentences with *they* and the readings times of sentences with *he*. However, the numerical trend exhibited by the *all*-sentences appears to provide some support that readers may have pursued a plural, referential interpretation of *they* in the all condition. In Regions 7 and 8, although the difference was not statistically significant, the *all*-sentences with *they* had a slower reading time than the *every*- and *each*-sentences. Recall that we found that in Region 8, the *all*-sentences overall had significantly slower reading times than the *every*-sentences. This effect is most likely driven by the numerical trend that the *all*-sentences with *they* were read the slowest. This pattern of reading times is consistent with *they* being interpreted as plural in the *all*-sentences but singular in the *every*- and *each*-sentences.

To test the hypothesis that *they* in the all condition was initially taken as a plural referential pronoun before encountering the disambiguating region (*only one*), we need to compare sentences with *all* as an antecedent quantifier to ones with a definite plural antecedent. In the definite plural sentences, we expect that readers can very easily take *they* as a plural referential pronoun. Thus, a processing difficulty should be attested in the singular region, where a clash would occur between plural *they* and *only one*. If *they* in the all condition is likewise interpreted as plural, then the processing profile of the *all*-sentences should pattern with the definite plural sentences, and not with the *every*/*each*-sentences. This is tested in Experiment 4.

## 5. Experiment 4: Self-paced Reading, Gendered Antecedent

Experiment 4 used the same self-paced reading procedure as Experiment 3 with male gendered antecedents. This experiment compared definite plural antecedents for *they* against quantified antecedents (*all, each*). The change to gendered antecedents was made to remove the possible confound discussed in Experiment 3.

### 5.1. Methods

#### 5.1.1. Participants

A total of 180 native English speakers (1 agender, 86 women, 87 men, and 6 non-binary) were recruited through Prolific and completed the experiment on Ibex Farm (Drummond, 2013). Participants range in age from 18 to 70 years, with a mean age of 36.79 years. Participants were compensated £2.50 upon confirmation of experiment completion.

#### 5.1.2. Task, Design, and Materials

A total of 36 male gendered nouns were used in this experiment. The first region of interest, the pronoun region (Region 3), is where the pronoun (*he/they*) is introduced. The second region of interest, the singular region (Region 7), remained constant for all items (*only one*) to confirm that the pronoun from Region 3 received a singular interpretation. An example test item set is given in (15).


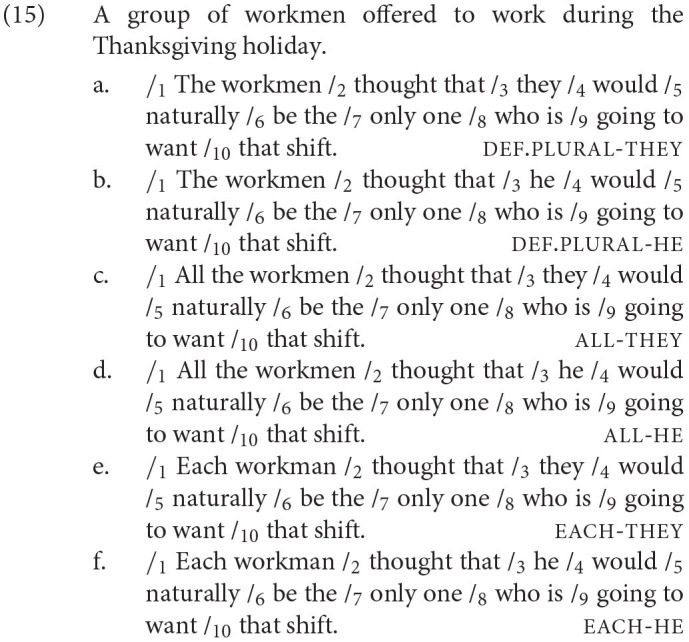


In addition to these test items, 36 sentences from a separate experiment on resumptive pronouns were included as filler items.

#### 5.1.3. Procedure

The procedure of Experiment 4 was identical to Experiment 3. Similarly, in Experiment 3, 36 test items were distributed across six lists in a Latin-Square design. Each list containing 36 test items and 36 fillers was randomized and evenly presented in either forward or reverse order. The comprehension question after each trial referenced the context sentence so that the interpretation of the target sentence would not influence responses. The corresponding comprehension question to the item in (15) is provided in (16).


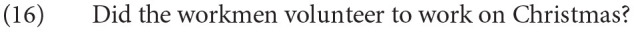


Two practice items were presented at the beginning of the experiment to familiarize participants with the task. After the experiment, participants were instructed to complete a demographic survey identical to the one presented in previous experiments. Participants were also required to confirm their Prolific ID to receive compensation.

### 5.2. Predictions

As with Experiment 3, if *they* is initially predisposed to forming a dependency with a plural antecedent, then the *each*-sentences should incur more processing costs than the definite plural or the *all*-sentences in the pronoun region and/or its spillover region. If *they* is underspecified, then there should be no difference among the tested antecedent types in the processing profile of *they*.

If *they* is interpreted as a plural referential pronoun, processing costs should be incurred in the they condition in the singular region and/or its spillover region. But if it is interpreted as a singular bound pronoun, no processing costs should be detected. The less distributive the antecedent noun phrase is, the more likely that *they* is interpreted as a plural referential pronoun, resulting in longer reading times. We thus expect the definite plural sentences to exhibit the longest reading times, followed by the *all*-sentences and then the *each*-sentences. In the he condition, there should be no difficulty with any of the antecedent noun phrases, as there is no number clash between singular *he* and *only one*.

### 5.3. Results

Just as in Experiment 3, we eliminated participants from the analysis whose range of mean reading times by region was less than 50 ms. This resulted in the removal of 27 participants. In addition, reading times of a region that were 10 SDs above the mean were removed, resulting in the further removal of 0.07% of the observations from the data.

The grand mean comprehension question response score on test sentences was 90%. The mean proportions of correct responses for the comprehension questions reported in Table 6 show that the manipulated factors had no impact on comprehension.

**Table 6:** The proportion of correct responses (SE), Experiment 4.

	**HE**	**THEY**
def.plural	0.91 (0.003)	0.91 (0.003)
all	0.90 (0.004)	0.91 (0.003)
each	0.91 (0.003)	0.89 (0.004)

**Figure 4:**
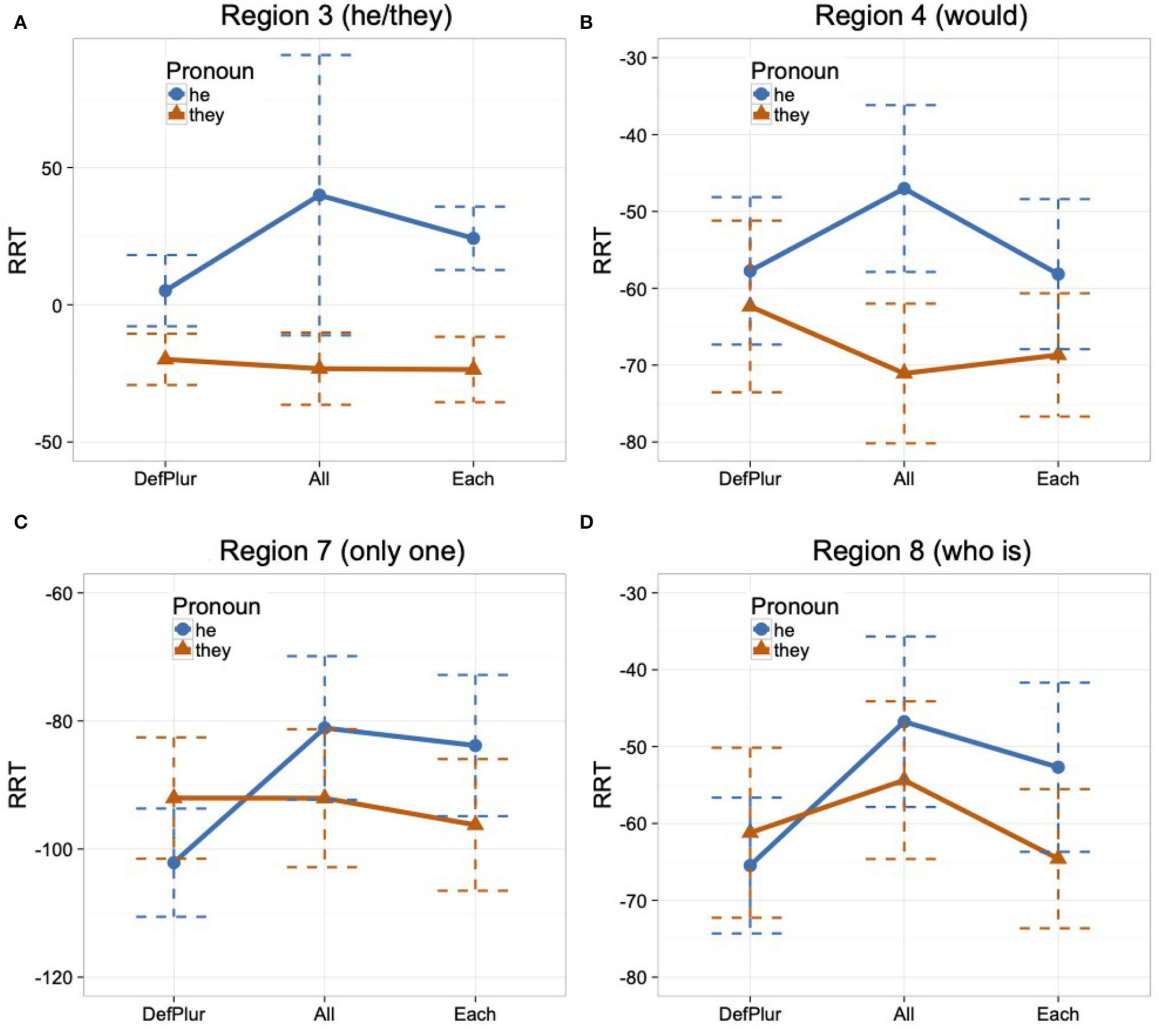
Mean RRTs by the condition in Regions 3, 4, 7, and 8 with standard errors **(A–D)**, Experiment 4.

Mean raw reading times by condition for each region (excluding the first and the last region) are reported in Table 7. Mean RRTs by condition for the first region of interest (Region 3), the pronoun region, and its spillover region (Region 4), as well as the second region of interest (Region 7), the singular region, and its spillover region (Region 8), are summarized in Figure 4.

**Table 7:** Mean raw reading times (SE) in ms, Experiment 4.

**Region**		**2**	**3**	**4**	**5**	**6**	**7**	**8**	**9**
def.plural	they	391 (7)	336 (5)	308 (7)	311 (6)	344 (24)	322 (6)	324 (7)	364 (7)
	he	377 (6)	332 (7)	312 (5)	315 (6)	317 (6)	311 (5)	319 (5)	370 (7)
all	they	404 (8)	332 (7)	299 (5)	306 (5)	314 (5)	322 (6)	330 (6)	374 (8)
	he	422 (9)	367 (27)	323 (6)	325 (6)	325 (6)	332 (6)	338 (6)	388 (8)
each	they	395 (7)	332 (7)	301 (5)	312 (9)	334 (21)	317 (6)	320 (6)	396 (26)
	he	417 (8)	351 (6)	312 (5)	322 (6)	334 (7)	330 (6)	332 (6)	381 (7)

As in the analysis performed in Experiment 3, we analyzed each region's RRTs with a mixed model, with a random-effects structure as described in Experiment 3, and fixed factors of Antecedent and Pronoun. Antecedent was forward-difference coded such that def.plural was compared to all (Antecedent1), and all was compared to each (Antecedent2). Pronoun was sum coded: he was coded as 1, and they was coded as –1.

In Region 3 (the pronoun region), the analysis revealed a main effect of Pronoun such that the he condition was read slower than the they condition regardless of antecedent type, as shown in Table 8^11^. The analysis of Region 4 (spillover region) revealed an interaction between Antecedent1 and Pronoun, but no interaction was found between Antecedent2 and Pronoun. Upon planned comparisons between the RRTs of the he and they conditions for definite plural and *all* antecedent types, a significant difference was found for the *all* comparison (Estimate = 11.37, *SE* = 3.14, *t* = 3.62, *p* < 0.01), with higher RRTs (slower reading time) in the he condition than the they condition. No difference was found for the definite plural comparison (Estimate = 2.54, *SE* = 3.55, *t* = 0.72, *p* = 0.47). The findings from the planned comparisons and the finding of no interaction between Antecedent2 and Pronoun taken together indicate that the he condition had significantly slower reading times than the they condition for both *all* and *each*, and to the same extent for both antecedent types.

**Table 8:** Fixed effects, Experiment 4, Regions 3, 4, 7, and 8.

**Region 3**	**Estimate**	**std. Error**	**df**	***t*-value**	**Pr(>|t|)**
(Intercept)	0.4265	7.0780	72.7692	0.060	0.952116
Antecedent1	–15.1496	19.5797	77.4217	–0.774	0.441441
Antecedent2	7.5992	18.1226	53.8070	0.419	0.676649
Pronoun1	22.4718	6.2562	55.2460	3.592	0.000699^***^
Antecedent1:Pronoun1	–18.7887	11.6092	5325.1917	–1.618	0.105629
Antecedent2:Pronoun1	6.7366	11.6113	5327.2138	0.580	0.561819
**Region 4**	**Estimate**	**std. Error**	**df**	**t value**	**Pr(**>|**t**|**)**
(Intercept)	–60.907	6.091	153.836	–10.000	<2e-16^***^
Antecedent1	–1.388	6.684	41.869	–0.208	0.8365
Antecedent2	5.065	7.845	37.163	0.646	0.5225
Pronoun1	6.338	3.322	41.030	1.908	0.0634^+^
Antecedent1:Pronoun1	–8.732	4.400	5085.662	–1.984	0.0473^*^
Antecedent2:Pronoun1	6.231	4.403	5084.378	1.415	0.1571
**Region 7**	**Estimate**	**std. Error**	**df**	**t value**	**Pr(**>|**t**|**)**
(Intercept)	–91.131	5.830	124.296	–15.631	<2e-16^***^
Antecedent1	–11.110	7.173	37.252	–1.549	0.1299
Antecedent2	3.697	9.031	37.067	0.409	0.6846
Pronoun1	1.998	3.316	35.851	0.603	0.5506
Antecedent1:Pronoun1	–9.933	4.765	4943.047	–2.084	0.0372^*^
Antecedent2:Pronoun1	–0.857	4.767	4938.919	–0.180	0.8573
**Region 8**	**Estimate**	**std. Error**	**df**	**t value**	**Pr(**>|**t**|**)**
(Intercept)	–57.507	5.357	132.977	–10.735	<2e-16^***^
Antecedent1	–12.939	6.496	50.717	–1.992	0.0518^+^
Antecedent2	8.475	7.321	35.740	1.158	0.2547
Pronoun1	2.236	3.531	39.313	0.633	0.5303
Antecedent1:Pronoun1	–5.226	4.816	5117.916	–1.085	0.2779
Antecedent2:Pronoun1	–2.476	4.816	5128.131	–0.514	0.6071
Formula in R: RRT ~ Antecedent*Pronoun + (1+Antecedent+Pronoun|Participant) + (1+Antecedent+Pronoun|Item)					

*Significance levels*:

*** p < 0.001, **p < 0.01,

* *p < 0.05*,

+ *p < 0.1*.

In Region 7 (the singular region), the analysis found an interaction between Antecedent1 and Pronoun, while no interaction between Antecedent2 and Pronoun was found, as shown in Table 8. Planned comparisons between the RRTs of the def.plural and all conditions for *they* and *he* pronoun types found no difference for the *they* comparison (Estimate = –0.69, *SE* = 3.38, *t* = –0.20, *p* = 0.84), but a significant difference was found for the *he* comparison (Estimate = –10.48, *SE* = 3.25, *t* = –3.22, *p* < 0.01). These findings taken together indicate that there was no difference in the processing profile of the *they*-sentences across antecedent types, but the *he*-sentences incurred processing costs in the all and each condition in comparison to the def.plural condition.

Region 8 (spillover region) did not reveal any interaction between antecedent type and pronoun type, nor a difference between pronoun types^12^.

### 5.4. Discussion

By comparing *they* with both singular and plural quantified antecedents, using a definite/referential plural antecedent as a baseline, we found no evidence that *they* is initially specified to form a dependency with a plural antecedent, just as in Experiment 3. That is, in the pronoun region (Region 3), there was no reading time difference among the tested antecedents (definite plural, *all* and *each*) in the they condition.

Interestingly, a difference did emerge between antecedent types in the spillover region (Region 4), in which the he condition was read slower than the they condition with quantified antecedents (*all* and *each*). No such difference arose when the noun phrase was a definite plural. One interpretation of this difference is as follows. There is no grammatical option where the pronoun *he* takes a definite plural as an antecedent, so readers might quickly accommodate a new, unheralded discourse referent for *he* in the spillover region. In the case of antecedents quantified by *each*, a dependency with *he* is a grammatical option, albeit potentially less preferred than a dependency with *they*, something that previous acceptability ratings studies have found (Han and Moulton, in press). The reading latencies in the spillover region could reflect a continued attempt to form a dependency between *he* and the quantified antecedent, but one that is more costly to make. Importantly, the all conditions patterned like each and not def.plural in this respect, perhaps suggesting that readers might attempt to integrate *he* with a quantified antecedent (plural or otherwise) and are less likely to have “moved on” to countenance an unheralded referent. We leave these speculations for further testing.

While it was entirely expected that *they* would be read faster than *he* in the *all*-sentences, given the obvious number differences, the same difference was found in the *each*-sentences. Taken together, these data suggest that not only does *they* not incur a number mismatch with a singular quantified antecedent, there is a general advantage for *they* over *he* as a bound variable with quantified antecedents. This is the case even when the antecedent nouns are male gendered, matching the gender feature of *he*.

As in Experiment 3, the singular region (Region 7, *only one*) was intended to serve as a “check” on whatever interpretation readers pursued for *they* before reaching this disambiguating point in the sentence. Our expectation was that reading time patterns in this region would tell us whether *they* was being interpreted as a singular bound variable or a plural referential pronoun. That is, we had expected to find, as a baseline, elevated reading times at this region in the def.plural condition with *they*, since we thought it likely that a referential plural interpretation would be most readily pursued in this case. This interpretation would be incompatible with the predicate *be the only one*. This is not what we found, however, and so we cannot interpret the effects in this region as planned. In particular, sentences with *they* were read uniformly quickly across all antecedent types, including def.plural. This is consistent with the underspecification approach—readers wait to adopt an interpretation for *they*, regardless of the number (singular/plural), quantificational nature (definite/quantified), and distributivity (*the*/*all*/*each*) of the antecedent. We acknowledge, however, that the data do not provide additional positive support for the underspecification approach. There were nevertheless effects in the singular region: the *he* sentences were read more slowly under quantifiers than under definite plurals. This could possibly reflect a general disadvantage for *he* as a bound variable as compared to *they*^13^.

## 6. General Discussion

Our goal in this article was to investigate whether the acceptability and processing of bound singular *they* are sensitive to the morphological number and the semantic distributivity of the antecedent quantifier, by comparing quantifier phrases headed by *all, every*, and *each*. If *they* initially prefers to form a dependency with a morphologically plural antecedent (Sanford and Filik, 2007; Van Handel et al., 2021)—and if this extends to bound variable uses of *they*—then we can expect to find a penalty when the antecedent phrase is headed by morphologically singular *every* or *each* in comparison to plural *all*. On the other hand, if the distributivity of the antecedent quantifier plays a role, we should see a cline in acceptability and/or processing ease among the tested antecedent quantifiers that mirrors the cline in distributivity from *all* to *every* to *each*.

In the self-paced reading studies (Experiments 3, 4), regardless of the gender of the antecedent, we found no evidence that bound singular *they* prefers a plural antecedent. Bound singular *they* with the morphologically singular quantifiers *every* and *each* was read no slower than with the morphologically plural *all*. In fact, *they* exhibited faster reading times than *he* with all the tested quantifiers. These results also bear on suggestive evidence in Han and Moulton (in press) that bound variable singular *they* may give rise to a weak number mismatch effect with singular quantified antecedents. However, the number of the antecedent was not directly manipulated in those studies. In the present studies, where the antecedent number *is* manipulated, we do not find such an effect.

As for the role of distributivity, while the acceptability studies (Experiments 1, 2) showed that bound singular *they* was preferred under more distributive quantifiers, the reading time measures from our self-paced reading studies did not detect any effects of antecedent quantifier distributivity. We did not find any differences in the processing profile of *they* among the tested quantifiers. In Experiment 3, the singular region exhibited no processing difficulty for *they* in comparison to singular *he* for *every* and *each*, as well as for *all*. In Experiment 4, while the *they*-sentences showed no hint of difficulty for any of the tested antecedent types (*all, each*, and definite plural), the *he*-sentences showed some difficulty for *all* and *each* in comparison to the definite plural.

These results taken together suggest that *they* is underspecified for number, which can be predicated by *only one* in sentences with either morphologically singular or plural quantifier antecedents, and that *they* has processing advantages over *he* as a bound variable.

Turning to the acceptability rating studies (Experiments 1, 2), we found that bound singular *they* is highly acceptable, regardless of the gender of the antecedent noun. The *each*-sentences were the most acceptable, followed by the *every*-sentences and then the *all*-sentences. These results show that bound singular *they* is not more acceptable with a morphologically plural antecedent quantifier; on the contrary, it is more acceptable with a morphologically singular antecedent quantifier. This is consistent with the findings from the reading times that showed no evidence of bound singular *they* being specified to prefer plural antecedents. But unlike the reading time measures, the acceptability ratings showed an effect of distributivity: the most distributive quantifier, *each*, had the highest acceptability ratings.

The finding that singular *they* bound by a singular quantifier poses no more difficulty nor a reduction in acceptability compared to singularly-interpreted *they* bound by a plural quantifier comports well with the proposals in the theoretical (Kratzer, 2009; Bjorkman, 2017; Konnelly and Cowper, 2020) and processing literature (Sanford et al., 2008) that *they* can be underspecified for number and gender features. As an underspecified pronoun, therefore, *they* should not clash with either singular or plural quantified antecedents, whether gendered or not. In fact, according to our reading time measures, not only did *they* easily retrieve singular quantified antecedents, *they* exhibited more processing ease than *he* for all the tested quantifiers. Thus, our online data further affirm the finding in previous offline studies that English users prefer *they* over singularly gendered pronouns (such as *he* or *she*) as bound variables, even with gendered antecedents (Camilliere et al., 2019; Conrod, 2019; Han and Moulton, in press).

Our findings stand to some extent in contrast with those in the literature regarding referential singular *they*, which has been reported to exhibit processing difficulties with singular antecedents (Sanford and Filik, 2007; Van Handel et al., 2021). Han and Moulton (in press) suggest that bound variable dependencies favor underspecified pronouns, whereas referential dependencies are less likely to, at least for more conservative speakers. We must remain cautious, however, in making comparisons between bound and referential singular *they*, as the latter is undergoing changes in the language that may impact both acceptability and processing. For instance, researchers have documented acceptability and processing differences relating to participants' exposure to referential singular *they* in non-binary accepting environments (Conrod, 2019; Chen et al., 2021). Future research should directly compare bound and referential singular *they* with attention to speaker variation in the case of referential *they*.

The underspecification approach is further bolstered by the differences between the acceptability rating studies and the reading time results. The online reading time measures did not detect any distributivity effects in the processing of bound singular *they*, though the offline acceptability ratings did. In particular, we found a cline of acceptability such that the each condition was rated highest, followed in turn by the every, all, and def.plural conditions. As we noted above, the reduction in acceptability can be traced to the need to interpret the *all*-sentences and the definite plural sentences distributively. This outcome is expected since distributive readings are generally dispreferred to collective ones (Frazier et al., 1999; Dotlačil and Brasoveanu, 2021).

The role of anaphora with *they* adds some further nuance to this picture. Repeating (5a) from above, we begin the discussion here with an example paralleling cases discussed in Rullmann (2003):


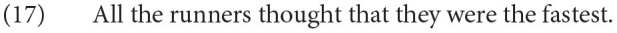


To obtain the plausible singularly-interpreted bound variable reading of (17), most researchers posit a distributive operator DIST (Rullmann, 2003; Sudo, 2014). The question is whether a collective interpretation is at all possible for (17), and when (and if) readers accommodate a distributive interpretation.

A collective interpretation would force a referential interpretation of *they*—where (17) perhaps describes a scenario in which the runners compare themselves as a group to a set of walkers—and does not require this operator. If this interpretation is possible, then the need to invoke a DIST operator is not clear on a first pass parse of (17). Indeed, it may only be upon later consideration of the sentence, and perhaps with other contextual information highlighting the bound variable reading, that a processor would even posit the DIST operator.

In our target items, the use of *the only one who* explicitly marks the embedded clause as a predicate over individuals. As discussed above, this forces participants toward a singular interpretation of *they*, but differences in antecedent quantifier distributivity, most crucially a distinction between *all* and *each*, only emerged as significant in the offline rating of sentences. We propose that this indicates the positing of the DIST operator is part of a later stage of computing the truth conditions of a sentence as a whole, which impacted sentence acceptability ratings^14^.

With an antecedent containing the quantifier *each*, a salient marker of distributivity, participants are primed for a predicate over individuals, and need not posit a covert operator. For the def.plural condition, the covert operator is the most likely way for the sentence to be felicitously resolved to a coherent meaning. That this is the condition rated significantly lowest in both Experiment 1 and Experiment 2 is not surprising: it is the one condition where additional structure (the DIST operator) must be assumed in the higher clause after the embedded clause is fully interpreted.

In the online study, *they* does appear to be processed with more difficulty in the def.plural condition relative to *he*. However, we believe that this appears more likely to be a property of some advantage for *he*, possibly arising from participants having already accommodated an unheralded antecedent for the more specified pronoun, as discussed above. If there were some impact of distributivity on *they* in this condition, we would expect this to manifest in a penalty on *they* in the def.plural condition relative to the other antecedents, and this is not what we observed. That we have not observed this in online processing speaks again to the underspecified nature of *they*: participants are not fixing on a plural interpretation of *they*, even when one is readily available. While it is true that other studies (Dotlačil and Brasoveanu, 2021) have found online evidence of a processing penalty when an antecedent is forced from a collective reading to a distributive one, these studies were targeting only the interpretation of an ambiguous sentential subject and its relationship to a verbal predicate, not the interpretation of a pronoun bound by that subject.

Keeping in mind that offline studies collect sentence acceptability judgments, measures of the sentence considered as a whole, the cline in ratings we have observed may be reflective of the different degrees to which a silent operator is necessary to arrive at a coherent interpretation of the sentence once it is considered in full. In the each condition, at most, a “floating” operation is necessary in order to have the quantifier in the right position to limit the domain of the verb to singleton sets. Nothing additional needs to be posited, and not surprisingly, this is the condition rated highest. The middle conditions, every and all, show an initially surprising behavior, not even being significantly differentiated in Experiment 2. This may be a reflection of participants' intuitions that the two quantifiers have some qualities in common with *each*, yet neither is its perfect equivalent. The lower ratings may not reflect so much difficulty (as in the def.plural condition) but more of a Gricean Maxim of Manner reaction. Participants are aware that a less ambiguous option is available (i.e., *each*), and maybe rate these middle cases slightly lower for this reason.

## 7. Conclusion

In the studies reported in this article, we investigated whether the processing and acceptability of bound singular *they* are sensitive to the morphological number and the semantic distributivity of the antecedent quantifier. Based on the findings from our reading time data and our acceptability rating data, we argue that (i) *they* is underspecified for number; and (ii) the construal mechanism of distributivity is part of a later stage of comprehension involving the computation of the truth conditions of a sentence.

## Data Availability Statement

The raw data supporting the conclusions of this article will be made available by the authors, without undue reservation.

## Ethics Statement

The studies involving human participants were reviewed and approved by Research Ethics Board, Simon Fraser University; Research Ethics Board, University of Toronto. The patients/participants provided their written informed consent to participate in this study.

## Author Contributions

C-hH and KM designed the experiments. C-hH, KM, TB, HG, DS, JW, and SW constructed the stimuli and wrote the manuscript. TB, HG, JW, and SW programmed the experiments and collected data. C-hH performed statistical analysis. All authors contributed to the article and approved the submitted version.

## Funding

This study was supported in part by Social Sciences and Humanities Research Council (SSHRC) Insight Grant 435-2014-0161 and Academy of Korean studies Grant AKS-2016-LAB-2250004 to C-hH, as well as SSHRC Insight Grant 435-2018-1012 to KM.

## Conflict of Interest

The authors declare that the research was conducted in the absence of any commercial or financial relationships that could be construed as a potential conflict of interest.

## Publisher's Note

All claims expressed in this article are solely those of the authors and do not necessarily represent those of their affiliated organizations, or those of the publisher, the editors and the reviewers. Any product that may be evaluated in this article, or claim that may be made by its manufacturer, is not guaranteed or endorsed by the publisher.

## Footnotes

^1^We use the term “gender” in the sense of “conceptual gender,” as described in Ackerman (2019, pp. 10), i.e., the gender “expressed, inferred, and used by a perceiver to classify a referent.” Linguistic expressions can be associated with conceptual gender, such as nouns like *sister* or *man* which are variably described in the literature as “definitionally gendered” (Kreiner et al., 2008; Ackerman, 2019, page 8), “lexically gendered” or “gender specific” (Konnelly and Cowper, 2020). In this article, we will describe these nouns as well as gender-stereotyped nouns (like *football player*) and proper names (*John*) simply as “gendered” nouns. Nouns that are associated with few or no gender stereotypes (such as *participants*) we call non-gendered.

^2^On a similar note, Sudo (2014)'s formal semantic analysis of singularly-interpreted *they* bound by plural *all* posits additional complexity as compared to *they* bound by *every*. This additional complexity could likewise counteract any advantage conferred by the morphological number congruency shared by *all* and *they*.

^3^For instance, while collective interpretations of *all* and *every* are possible in *Take all/every one/each one of these apples* (i.e., where all the apples are taken as a bushel at once), *each* requires a distributive reading (where individual apples are taken one-by-one).

^4^Participants in all experiments self-report their gender identity in an open text field. We aggregate those responses into common terms (e.g., collapsing “F”, “female,” and “woman” to a single category “woman”). The label “unclassified” describes a small number of participants who entered a value that is not typically a gender identity, such as a descriptor of sexual orientation, or, in one case, just the word “yes”.

^5^Antecedent1 was coded 3/4 for def.plural and the other levels were coded –1/4. Antecedent2 was coded 1/2 for def.plural and all, and –1/2 for every and each. Antecedent3 was coded 1/4 for def.plural, all and every, and –3/4 for each.

^6^Reviewers suggest two other possible reasons for the relatively high ratings for the definite plural cases. The first is that the plurality of the definite description may be semantically vacuous, following proposals in the theoretical semantic literature (Sauerland et al., 2005). On that view, it may be that definite plurals can support singular reference thus allowing for *they* to be interpreted singularly here. We leave this as a viable contender to our speculation regarding the ready availability of a distributive operator. We note, however, that the all conditions also contained a plural (definite) component (e.g., *all the cyclists*) and more favorably anteceded singular *they* than the bare definite plural, suggesting that quantification (and by extension distributivity) plays a role. A second alternative is that readers arrive at a collective reading and simply assume that *-s* is missing from *one*. This is not a possibility we can resolve without further testing, though even if participants did adopt this strategy, it would not be incompatible with a DIST operator, as *ones* itself would be ambiguous between a distributive or collective reading.

^7^The experiment was set up in Prolific to specifically exclude any participant ID's that had participated in Experiment 1 from participating in Experiment 2. These exclusions were carried forward for Experiments 3 and 4 to ensure no participant who had participated in a previous experiment could participate in subsequent experiments reported in this article.

^8^Most of the non-gendered nouns we used in Experiment 1 were independently rated as gender-neutral by Doherty and Conklin (2017). The remainder were determined as such based on the authors' intuitions. The gendered nouns used in Experiment 2 were all chosen based on the authors' intuitions. As suggested by a reviewer, we sought to verify our intuitions with a norming task in which 17 participants (who did not participate in Experiments 1–4) were asked to select “female,” “neutral,” or “male” for each of the antecedent nouns we used in the experiments reported in this article. The proportion of “male” selection was 0.92 for the nouns we classified as gendered, and the proportion of “neutral” selection was 0.87 for the nouns we classified as non-gendered. Pearson's chi-squared test revealed that the participants' selections were significantly associated with whether or not a noun was gendered (χ^2^ = 877, df = 2, *p* < 0.001). Pearson residuals of each selection grouped by noun gender showed that the “male” selection is positively associated with gendered nouns and negatively associated with non-gendered nouns, whereas the “neutral” selection is positively associated with non-gendered nouns and negatively associated with gendered nouns. These results support the classification of nouns in Experiment 1 as non-gendered and those in Experiment 2 as male-gendered.

^9^As suggested by a reviewer, we conducted a preliminary analysis to compare the results of Experiment 1 and Experiment 2 directly by including the antecedent noun gender as a fixed factor. According to the analysis, there was no effect of the gender of the antecedent noun. We found that regardless of whether the antecedent noun was gendered or not, the z-transformed mean rating of def.plural was significantly lower than the rating of all (Est = –0.25, *SE* = 0.05, *t* = –4.79, *p* < 0.001), which was significantly lower than the rating of every (Est = –0.11, *SE* = 0.05, *t* = –2.33, *p* < 0.05), which was in turn significantly lower than the rating of each (Est = –0.15, *SE* = 0.04, *t* = –4.19, *p* < 0.001).

^10^The mean raw reading times summarized in Table 4 show a numerical trend opposite from the one of the RRTs in Region 3 such that the they condition exhibits higher mean raw reading times than the he condition for all antecedent types. We think this trend is an artifact of the longer character length of *they* in comparison to *he*, which is reversed by the RRT transformation. Importantly, no statistical difference was found between the raw reading times of the they and he conditions, and thus, we have no evidence that the they condition was read slower than the he condition from the raw reading time data.

^11^In Region 3, the raw mean reading times for the they condition are numerically higher than for the he condition in the definite plural sentences. This is the opposite trend from the RRTs, which take character length into consideration. The raw reading time difference between the two pronoun conditions in the definite plural sentences was not statistically significant, however, and thus we have no evidence that the they condition was read slower than the he condition. The raw reading times in the *all*- and *each*-sentences show the same trend as the RRTs.

^12^We conducted power analyses on the regions of analyses for Experiments 3 and 4, using the powerSim function in the simr package (Green et al., 2022). All power analyses returned over 89% power with the fixed effects set at the 0.05 significance level for the interaction terms. This suggests that both experiments had large enough sample sizes to detect an interaction effect if there was one in Regions 3, 4, 7, or 8.

^13^Suggested by a reviewer, we conducted a preliminary analysis directly comparing the results of Experiment 3 and Experiment 4 in the all and the each condition, the two conditions that were tested in both experiments, by including the antecedent noun gender as a fixed factor. We found a main effect of antecedent noun gender in Regions 7 and 8 (Region 7: Est = –23.23, *SE* = 5.51, *t* = –4.22, *p* < 0.001; Region 8: Est = –37.42, *SE* = 5.22, *t* = –7.16, *p* < 0.001) such that the non-gendered condition showed slower reading time than the gendered condition overall. We also found an interaction of the pronoun type and the antecedent noun gender in Region 7 (Est = –5.45, *SE* = 2.34, *t* = –2.33, *p* < 0.05) such that when the antecedent noun was gendered, the he condition showed slower reading time than the they condition for both types of quantifiers. Importantly, the antecedent noun gender did not interact in any way with the type of quantifiers. That is, the processing profile of the pronouns showed the same pattern across the all and the each condition within each experiment in all regions of analyses.

^14^A reviewer notes that such offline/online differences may alternatively relate to differences between deeper and shallower processing. That is if the sentence rating task prompts deeper processing, perhaps because the task allows participants to devote more attention to reflective consideration of the sentence, this could be why the effects of distributivity show up in the ratings but not self-paced reading. We leave this as an open question, as it would require follow-up studies that manipulate the depth of processing. We are likewise cautious because the relationship between so-called deep/shallow processing and anaphoric dependencies has been argued to be rather complex: Koorneef and Reuland (2016) suggest that anaphoric dependencies may sometimes prefer representations that implicate deeper processing.
