# Supplementary material for: Bound Variable Singular They Is Underspecified: The Case of All vs. Every
Source: Front Psychol. 2022 Jun 2;13:880687. doi: 10.3389/fpsyg.2022.880687 (PMC9201828; doi:10.3389/fpsyg.2022.880687)
Supplement: Supplementary file 1 [file Data_Sheet_1.PDF]

Supplementary Material

All test materials used for the experiments are provided below.

1 EXPERIMENT 1 TEST MATERIALS: RATING, NON-GENDERED ANTECEDENT

1. Outside the school, there was a group of students talking about how the exam went.
   a. The students thought that they were the only one who got an A plus.
   b. All the students thought that they were the only one who got an A plus.
   c. Every student thought that they were the only one who got an A plus.
   d. Each student thought that they were the only one who got an A plus.
2. At the racing track early in the morning, a bunch of runners were bragging about race times.
   a. The runners said that they were the only one who could beat the record.
   b. All the runners said that they were the only one who could beat the record.
   c. Every runner said that they were the only one who could beat the record.
   d. Each runner said that they were the only one who could beat the record.
3. After the restaurant closed for the night, a group of servers were talking about tips.
   a. The servers thought that they were the only one who got really bad tips.
   b. All the servers thought that they were the only one who got really bad tips.
   c. Every server thought that they were the only one who got really bad tips.
   d. Each server thought that they were the only one who got really bad tips.
4. A group of freshmen had applied for a prestigious scholarship at the university.
   a. The freshmen believed that they were the only one who would get the award.
   b. All the freshmen believed that they were the only one who would get the award.
   c. Every freshman believed that they were the only one who would get the award.
   d. Each freshman believed that they were the only one who would get the award.
5. A bunch of chefs were competing to make the best cupcakes on a popular baking show.
   a. The chefs hoped that they were the only one who used chocolate frosting.
   b. All the chefs hoped that they were the only one who used chocolate frosting.
   c. Every chef hoped that they were the only one who used chocolate frosting.
   d. Each chef hoped that they were the only one who used chocolate frosting.
6. A group of teenagers were deciding on a video game to play at the house party.
   a. The teenagers believed that they were the only one who hated playing Mario Kart.
   b. All the teenagers believed that they were the only one who hated playing Mario Kart.
   c. Every teenager believed that they were the only one who hated playing Mario Kart.
   d. Each teenager believed that they were the only one who hated playing Mario Kart.
7. A big group of cyclists decided to go for a ride despite the poor weather.
   a. The cyclists thought that they were the only one who liked the pouring rain.
   b. All the cyclists thought that they were the only one who liked the pouring rain.

   c. Every cyclist thought that they were the only one who liked the pouring rain.
   d. Each cyclist thought that they were the only one who liked the pouring rain.
8. A group of baristas were competing in the final of the World Barista Championship.
   a. The baristas thought that they were the only one who made good latte art.
   b. All the baristas thought that they were the only one who made good latte art.
   c. Every barista thought that they were the only one who made good latte art.
   d. Each barista thought that they were the only one who made good latte art.
9. A bunch of kids made it to the TV showing of the national Spelling Bee.
   a. The kids thought that they were the only one who could spell the hardest word.
   b. All the kids thought that they were the only one who could spell the hardest word.
   c. Every kid thought that they were the only one who could spell the hardest word.
   d. Each kid thought that they were the only one who could spell the hardest word.
10. A group of interns had applied for one permanent position at the company.
   a. The interns expected that they were the only one who would get the job offer.
   b. All the interns expected that they were the only one who would get the job offer.
   c. Every intern expected that they were the only one who would get the job offer.
   d. Each intern expected that they were the only one who would get the job offer.
11. A group of workers volunteered to work during the Thanksgiving holiday.
   a. The workers thought that they were the only one who would want that shift.
   b. All the workers thought that they were the only one who would want that shift.
   c. Every worker thought that they were the only one who would want that shift.
   d. Each worker thought that they were the only one who would want that shift.
12. A group of salespeople were competing to win the employee of the year award.
   a. The salespeople hoped that they were the only one who could win the award.
   b. All the salespeople hoped that they were the only one who could win the award.
   c. Every salesperson hoped that they were the only one who could win the award.
   d. Each salesperson hoped that they were the only one who could win the award.
13. A group of commuters were waiting on the train platform at rush hour.
   a. The commuters thought that they were the only one who would get a seat.
   b. All the commuters thought that they were the only one who would get a seat.
   c. Every commuter thought that they were the only one who would get a seat.
   d. Each commuter thought that they were the only one who would get a seat.
14. A bunch of spectators turned out early to see the parade through the city.
   a. The spectators thought that they were the only one who had a good view.
   b. All the spectators thought that they were the only one who had a good view.
   c. Every spectator thought that they were the only one who had a good view.
   d. Each spectator thought that they were the only one who had a good view.
15. At the park, a group of neighbors met to compare gardening plans for next year.

a. The neighbors thought that they were the only one who would plant tulips.
b. All the neighbors thought that they were the only one who would plant tulips.
c. Every neighbor thought that they were the only one who would plant tulips.
d. Each neighbor thought that they were the only one who would plant tulips.

16. A group of customers rushed to get an exclusive discount on a product.
    a. The customers thought that they were the only one who would get a discount.
    b. All the customers thought that they were the only one who would get a discount.
    c. Every customer thought that they were the only one who would get a discount.
    d. Each customer thought that they were the only one who would get a discount.
17. A group of managers competed to get a raise for having the best performance.
    a. The managers thought that they were the only one who would get a raise.
    b. All the managers thought that they were the only one who would get a raise.
    c. Every manager thought that they were the only one who would get a raise.
    d. Each manager thought that they were the only one who would get a raise.
18. A group of performers sang for a live audience to win a prize.
    a. The performers thought that they were the only one who would get a prize.
    b. All the performers thought that they were the only one who would get a prize.
    c. Every performer thought that they were the only one who would get a prize.
    d. Each performer thought that they were the only one who would get a prize.
19. At a family reunion, a group of cousins were competing in a sack race.
    a. The cousins thought that they were the only one who would win the race.
    b. All the cousins thought that they were the only one who would win the race.
    c. Every cousin thought that they were the only one who would win the race.
    d. Each cousin thought that they were the only one who would win the race.
20. A group of fans had bet on different teams to win the championship.
    a. The fans thought that they were the only one who had chosen the winning team.
    b. All the fans thought that they were the only one who had chosen the winning team.
    c. Every fan thought that they were the only one who had chosen the winning team.
    d. Each fan thought that they were the only one who had chosen the winning team.
21. A group of directors had submitted short films to the local festival.
    a. The directors expected that they were the only one who would be featured on opening night.
    b. All the directors expected that they were the only one who would be featured on opening night.
    c. Every director expected that they were the only one who would be featured on opening night.
    d. Each director expected that they were the only one who would be featured on opening night.
22. At a conference, a group of mathematicians were arguing about the solution to a problem.
    a. The mathematicians said that they were the only one who had the correct solution.
    b. All the mathematicians said that they were the only one who had the correct solution.
    c. Every mathematician said that they were the only one who had the correct solution.

   d. Each mathematician said that they were the only one who had the correct solution.
23. A bunch of musicians were having a disagreement about who was in tune.
   a. The musicians said that they were the only one who was in tune.
   b. All the musicians said that they were the only one who was in tune.
   c. Every musician said that they were the only one who was in tune.
   d. Each musician said that they were the only one who was in tune.
24. A group of journalists were comparing sources used in the past month.
   a. The journalists thought that they were the only one who had a reliable source.
   b. All the journalists thought that they were the only one who had a reliable source.
   c. Every journalist thought that they were the only one who had a reliable source.
   d. Each journalist thought that they were the only one who had a reliable source.

2 EXPERIMENT 2 TEST MATERIALS: RATING, GENDERED ANTECEDENT

1. A group of boys were talking about how the exam went.
   a. The boys thought that they were the only one who got an A plus.
   b. All the boys thought that they were the only one who got an A plus.
   c. Every boy thought that they were the only one who got an A plus.
   d. Each boy thought that they were the only one who got an A plus.
2. A bunch of firemen were bragging about who was the fastest.
   a. The firemen said that they were the only one who could beat the record.
   b. All the firemen said that they were the only one who could beat the record.
   c. Every fireman said that they were the only one who could beat the record.
   d. Each fireman said that they were the only one who could beat the record.
3. A group of busboys were talking about tips at the restaurant.
   a. The busboys thought that they were the only one who got really bad tips.
   b. All the busboys thought that they were the only one who got really bad tips.
   c. Every busboy thought that they were the only one who got really bad tips.
   d. Each busboy thought that they were the only one who got really bad tips.
4. A group of men had applied for a prestigious scholarship at the university.
   a. The men believed that they were the only one who would get the award.
   b. All the men believed that they were the only one who would get the award.
   c. Every man believed that they were the only one who would get the award.
   d. Each man believed that they were the only one who would get the award.
5. A bunch of dads were competing to make the best cupcakes on a popular baking show.
   a. The dads hoped that they were the only one who used chocolate frosting.
   b. All the dads hoped that they were the only one who used chocolate frosting.
   c. Every dad hoped that they were the only one who used chocolate frosting.
   d. Each dad hoped that they were the only one who used chocolate frosting.
6. A group of guys were deciding on a video game to play at the house party.

a. The guys believed that they were the only one who hated playing Mario Kart.
b. All the guys believed that they were the only one who hated playing Mario Kart.
c. Every guy believed that they were the only one who hated playing Mario Kart.
d. Each guy believed that they were the only one who hated playing Mario Kart.

7. A big group of cowboys decided to go for a ride despite the poor weather.
   a. The cowboys thought that they were the only one who liked the pouring rain.
   b. All the cowboys thought that they were the only one who liked the pouring rain.
   c. Every cowboy thought that they were the only one who liked the pouring rain.
   d. Each cowboy thought that they were the only one who liked the pouring rain.
8. A group of strongmen were competing in the final of the weightlifting championship.
   a. The strongmen thought that they were the only one who broke a new record.
   b. All the strongmen thought that they were the only one who broke a new record.
   c. Every strongman thought that they were the only one who broke a new record.
   d. Each strongman thought that they were the only one who broke a new record.
9. A bunch of schoolboys made it to the TV showing of the national Spelling Bee.
   a. The schoolboys thought that they were the only one who could spell the hardest word.
   b. All the schoolboys thought that they were the only one who could spell the hardest word.
   c. Every schoolboy thought that they were the only one who could spell the hardest word.
   d. Each schoolboy thought that they were the only one who could spell the hardest word.
10. A group of handymen had applied for one permanent position at the new shop.
   a. The handymen expected that they were the only one who would get the job offer.
   b. All the handymen expected that they were the only one who would get the job offer.
   c. Every handyman expected that they were the only one who would get the job offer.
   d. Each handyman expected that they were the only one who would get the job offer.
11. A group of workmen offered to work during the Thanksgiving holiday.
   a. The workmen thought that they were the only one who would want that shift.
   b. All the workmen thought that they were the only one who would want that shift.
   c. Every workman thought that they were the only one who would want that shift.
   d. Each workman thought that they were the only one who would want that shift.
12. A group of salesmen were competing to win the employee of the year award.
   a. The salesmen hoped that they were the only one who could win the award.
   b. All the salesmen hoped that they were the only one who could win the award.
   c. Every salesman hoped that they were the only one who could win the award.
   d. Each salesman hoped that they were the only one who could win the award.
13. A group of businessmen were waiting on the train platform at rush hour.
   a. The businessmen thought that they were the only one who would get a seat.
   b. All the businessmen thought that they were the only one who would get a seat.
   c. Every businessman thought that they were the only one who would get a seat.

d. Each businessman thought that they were the only one who would get a seat.

14. A bunch of fathers turned out early to save spots to view the parade through the city.
    a. The fathers thought that they were the only one who had a good view.
    b. All the fathers thought that they were the only one who had a good view.
    c. Every father thought that they were the only one who had a good view.
    d. Each father thought that they were the only one who had a good view.
15. A group of grandfathers met at the park to compare gardening plans for next year.
    a. The grandfathers thought that they were the only one who would plant tulips.
    b. All the grandfathers thought that they were the only one who would plant tulips.
    c. Every grandfather thought that they were the only one who would plant tulips.
    d. Each grandfather thought that they were the only one who would plant tulips.
16. A group of young brothers rushed to get an exclusive discount on a new video game.
    a. The brothers thought that they were the only one who would get the game.
    b. All the brothers thought that they were the only one who would get the game.
    c. Every brother thought that they were the only one who would get the game.
    d. Each brother thought that they were the only one who would get the game.
17. A bunch of sons were hoping to take over the family autoshop.
    a. The sons thought that they were the only one who would be the next shop owner.
    b. All the sons thought that they were the only one who would be the next shop owner.
    c. Every son thought that they were the only one who would be the next shop owner.
    d. Each son thought that they were the only one who would be the next shop owner.
18. A group of baritones sang for a live audience to win a prize.
    a. The baritones thought that they were the only one who would get a prize.
    b. All the baritones thought that they were the only one who would get a prize.
    c. Every baritone thought that they were the only one who would get a prize.
    d. Each baritone thought that they were the only one who would get a prize.
19. A group of uncles were competing in a sack race at a family reunion.
    a. The uncles thought that they were the only one who would win the race.
    b. All the uncles thought that they were the only one who would win the race.
    c. Every uncle thought that they were the only one who would win the race.
    d. Each uncle thought that they were the only one who would win the race.
20. A group of linesmen had bet on different teams to win the championship.
    a. The linesmen thought that they were the only one who had chosen the winning team.
    b. All the linesmen thought that they were the only one who had chosen the winning team.
    c. Every linesman thought that they were the only one who had chosen the winning team.
    d. Each linesman thought that they were the only one who had chosen the winning team.
21. A group of cameramen had submitted short films to the local festival.
    a. The cameramen expected that they were the only one who would be featured on opening night.

b. All the cameramen expected that they were the only one who would be featured on opening night.
c. Every cameraman expected that they were the only one who would be featured on opening night.
d. Each cameraman expected that they were the only one who would be featured on opening night.

22. A bunch of dukes were talking about their future inheritance.
    a. The dukes hoped that they were the only one who would inherit a large tract of land.
    b. All the dukes hoped that they were the only one who would inherit a large tract of land.
    c. Every duke hoped that they were the only one who would inherit a large tract of land.
    d. Each duke hoped that they were the only one who would inherit a large tract of land.
23. A group of weathermen were discussing the latest climate change research.
    a. The weathermen believed that they were the only one who would understand the research.
    b. All the weathermen believed that they were the only one who would understand the research.
    c. Every weatherman believed that they were the only one who would understand the research.
    d. Each weatherman believed that they were the only one who would understand the research.
24. A group of fishermen were comparing catches from the last summer.
    a. The fishermen thought that they were the only one who was not exaggerating the size of the catch.
    b. All the fishermen thought that they were the only one who was not exaggerating the size of the catch.
    c. Every fisherman thought that they were the only one who was not exaggerating the size of the catch.
    d. Each fisherman thought that they were the only one who was not exaggerating the size of the catch.

3 EXPERIMENT 3 TEST MATERIALS: SELF-PACED READING, NON-GENDERED ANTECEDENT

1. Outside the school, there was a group of students talking about how the exam went.
   a. All the students / thought that / [they/he] / would / definitely / be the / only one / who would / get / an A plus.
   b. Every student / thought that / [they/he] / would / definitely / be the / only one / who would / get / an A plus.
   c. Each student / thought that / [they/he] / would / definitely / be the / only one / who would / get / an A plus.
2. At the racing track early in the morning a bunch of runners were bragging.
   a. All the runners / said that / [they/he] / would / certainly / be the / only one / who could / beat / the record.
   b. Every runner / said that / [they/he] / would / certainly / be the / only one / who could / beat / the record.
   c. Each runner / said that / [they/he] / would / certainly / be the / only one / who could / beat / the record.
3. After the restaurant closed for the night a group of servers were talking.
   a. All the servers / thought that / [they/he] / would / probably / be the / only one / who would / get / bad tips.

   b. Every server / thought that / [they/he] / would / probably / be the / only one / who would / get / bad tips.
   c. Each server / thought that / [they/he] / would / probably / be the / only one / who would / get / bad tips.

4. A group of freshmen had applied for a prestigious scholarship at the university.
   a. All the freshmen / believed that / [they/he] / would / rightfully / be the / only one / who would / get / the award.
   b. Every freshman / believed that / [they/he] / would / rightfully / be the / only one / who would / get / the award.
   c. Each freshman / believed that / [they/he] / would / rightfully / be the / only one / who would / get / the award.
5. A bunch of chefs were competing to make the best cake on a popular baking show.
   a. All the chefs / thought that / [they/he] / might / luckily / be the / only one / who would / use / chocolate frosting.
   b. Every chef / thought that / [they/he] / might / luckily / be the / only one / who would / use / chocolate frosting.
   c. Each chef / thought that / [they/he] / might / luckily / be the / only one / who would / use / chocolate frosting.
6. A group of teenagers were deciding on a video game to play at the house party.
   a. All the teenagers / believed that / [they/he] / would / surprisingly / be the / only one / who would / suggest / Mario Kart.
   b. Every teenager / believed that / [they/he] / would / surprisingly / be the / only one / who would / suggest / Mario Kart.
   c. Each teenager / believed that / [they/he] / would / surprisingly / be the / only one / who would / suggest / Mario Kart.
7. A big group of cyclists decided to go for a ride despite the poor weather.
   a. All the cyclists / thought that / [they/he] / would / surely / be the / only one / who would / like / the rain.
   b. Every cyclist / thought that / [they/he] / would / surely / be the / only one / who would / like / the rain.
   c. Each cyclist / thought that / [they/he] / would / surely / be the / only one / who would / like / the rain.
8. A group of baristas were competing in the final of the World Barista Championship.
   a. All the baristas / thought that / [they/he] / would / naturally / be the / only one / who could / make / good latte art.
   b. Every barista / thought that / [they/he] / would / naturally / be the / only one / who could / make / good latte art.
   c. Each barista / thought that / [they/he] / would / naturally / be the / only one / who could / make / good latte art.
9. A bunch of kids made it to the TV showing of the national Spelling Bee.
   a. All the kids / thought that / [they/he] / might / ultimately / be the / only one / who could / spell / the hardest word.

b. Every kid / thought that / [they/he] / might / ultimately / be the / only one / who could / spell / the hardest word.

c. Each kid / thought that / [they/he] / might / ultimately / be the / only one / who could / spell / the hardest word.

10. A group of interns had applied for one permanent position at the company.

a. All the interns / expected that / [they/he] / would / definitely / be the / only one / who would / get / an offer.

b. Every intern / expected that / [they/he] / would / definitely / be the / only one / who would / get / an offer.

c. Each intern / expected that / [they/he] / would / definitely / be the / only one / who would / get / an offer.

11. A group of workers volunteered to work during the Thanksgiving holiday.

a. All the workers / thought that / [they/he] / would / naturally / be the / only one / who would / want / that shift.

b. Every worker / thought that / [they/he] / would / naturally / be the / only one / who would / want / that shift.

c. Each worker / thought that / [they/he] / would / naturally / be the / only one / who would / want / that shift.

12. A group of salespeople were competing to win a prestigious award.

a. All the salespeople / hoped that / [they/he] / might / actually / be the / only one / who could / win / the award.

b. Every salesperson / hoped that / [they/he] / might / actually / be the / only one / who could / win / the award.

c. Each salesperson / hoped that / [they/he] / might / actually / be the / only one / who could / win / the award.

13. A group of commuters were waiting on the train platform at rush hour.

a. All the commuters / thought that / [they/he] / would / fortunately / be the / only one / who would / get / a seat.

b. Every commuter / thought that / [they/he] / would / fortunately / be the / only one / who would / get / a seat.

c. Each commuter / thought that / [they/he] / would / fortunately / be the / only one / who would / get / a seat.

14. A bunch of spectators turned out early to see the parade through the city.

a. All the spectators / thought that / [they/he] / would / definitely / be the / only one / who would / get / a view.

b. Every spectator / thought that / [they/he] / would / definitely / be the / only one / who would / get / a view.

c. Each spectator / thought that / [they/he] / would / definitely / be the / only one / who would / get / a view.

15. At the park a group of neighbors met to talk about gardening for the next year.

a. All the neighbors / thought that / [they/he] / would / likely / be the / only one / who would / plant / more tulips.

b. Every neighbor / thought that / [they/he] / would / likely / be the / only one / who would / plant / more tulips.

c. Each neighbor / thought that / [they/he] / would / likely / be the / only one / who would / plant / more tulips.

16. A group of customers rushed to get an exclusive discount on a product.

a. All the customers / thought that / [they/he] / might / possibly / be the / only one / who would / get / a discount.

b. Every customer / thought that / [they/he] / might / possibly / be the / only one / who would / get / a discount.

c. Each customer / thought that / [they/he] / might / possibly / be the / only one / who would / get / a discount.

17. A group of managers competed to get a raise for having the best performance.

a. All the managers / thought that / [they/he] / would / likely / be the / only one / who would / get / a raise.

b. Every manager / thought that / [they/he] / would / likely / be the / only one / who would / get / a raise.

c. Each manager / thought that / [they/he] / would / likely / be the / only one / who would / get / a raise.

18. A group of performers sang at a live concert to win a prize.

a. All the performers / thought that / [they/he] / would / probably / be the / only one / who would / get / a prize.

b. Every performer / thought that / [they/he] / would / probably / be the / only one / who would / get / a prize.

c. Each performer / thought that / [they/he] / would / probably / be the / only one / who would / get / a prize.

19. At a reunion a group of cousins were competing in a sack race.

a. All the cousins / thought that / [they/he] / would / surely / be the / only one / who would / win / the race.

b. Every cousin / thought that / [they/he] / would / surely / be the / only one / who would / win / the race.

c. Each cousin / thought that / [they/he] / would / surely / be the / only one / who would / win / the race.

20. A group of fans were betting on the championship game.

a. All the fans / thought that / [they/he] / might / ultimately / be the / only one / who would / choose / the winners.

b. Every fan / thought that / [they/he] / might / ultimately / be the / only one / who would / choose / the winners.

c. Each fan / thought that / [they/he] / might / ultimately / be the / only one / who would / choose / the winners.

21. A group of amateurs had submitted work to a local festival.

a. All the amateurs / expected that / [they/he] / would / actually / be the / only one / who would / attend / the festival.

b. Every amateur / expected that / [they/he] / would / actually / be the / only one / who would / attend / the festival.

c. Each amateur / expected that / [they/he] / would / actually / be the / only one / who would / attend / the festival.

22. At a conference a group of mathematicians were arguing about the solution to a problem.

a. All the mathematicians / said that / [they/he] / would / certainly / be the / only one / who would / get / the solution.

b. Every mathematician / said that / [they/he] / would / certainly / be the / only one / who would / get / the solution.

c. Each mathematician / said that / [they/he] / would / certainly / be the / only one / who would / get / the solution.

23. A bunch of musicians were having a disagreement about tuning.

a. All the musicians / said that / [they/he] / would / probably / be the / only one / who had / produced / the right note.

b. Every musician / said that / [they/he] / would / probably / be the / only one / who had / produced / the right note.

c. Each musician / said that / [they/he] / would / probably / be the / only one / who had / produced / the right note.

24. A group of journalists were comparing information used in the past month.

a. All the journalists / thought that / [they/he] / might / possibly / be the / only one / who had / gotten / reliable sources.

b. Every journalist / thought that / [they/he] / might / possibly / be the / only one / who had / gotten / reliable sources.

c. Each journalist / thought that / [they/he] / might / possibly / be the / only one / who had / gotten / reliable sources.

25. A group of travelers were waiting to get on a plane for a very long flight.

a. All the travelers / believed that / [they/he] / would / actually / be the / only one / who would / enjoy / the flight.

b. Every traveler / believed that / [they/he] / would / actually / be the / only one / who would / enjoy / the flight.

c. Each traveler / believed that / [they/he] / would / actually / be the / only one / who would / enjoy / the flight.

26. At the airport a bunch of passengers were at the gate waiting for the boarding call.

a. All the passengers / believed that / [they/he] / would / probably / be the / only one / who would / get / an upgrade.

b. Every passenger / believed that / [they/he] / would / probably / be the / only one / who would / get / an upgrade.

c. Each passenger / believed that / [they/he] / would / probably / be the / only one / who would / get / an upgrade.

27. A group of tenants were gossiping while doing laundry.
    a. All the tenants / said that / [they/he] / might / actually / be the / only one / who had / found / a nice landlord.
    b. Every tenant / said that / [they/he] / might / actually / be the / only one / who had / found / a nice landlord.
    c. Each tenant / said that / [they/he] / might / actually / be the / only one / who had / found / a nice landlord.
28. A group of vacationers were arguing about how to get downtown.
    a. All the vacationers / thought that / [they/he] / would / definitely / be the / only one / who could / find / the fastest route.
    b. Every vacationer / thought that / [they/he] / would / definitely / be the / only one / who could / find / the fastest route.
    c. Each vacationer / thought that / [they/he] / would / definitely / be the / only one / who could / find / the fastest route.
29. At the orchard a group of apple pickers were competing to pick the most fruit.
    a. All the apple pickers / thought that / [they/he] / would / surely / be the / only one / who would / fill / a dozen baskets.
    b. Every apple picker / thought that / [they/he] / would / surely / be the / only one / who would / fill / a dozen baskets.
    c. Each apple picker / thought that / [they/he] / would / surely / be the / only one / who would / fill / a dozen baskets.
30. A group of young artists were competing for a prestigious painting scholarship.
    a. All the artists / believed that / [they/he] / would / surely / be the / only one / who would / receive / the scholarship.
    b. Every artist / believed that / [they/he] / would / surely / be the / only one / who would / receive / the scholarship.
    c. Each artist / believed that / [they/he] / would / surely / be the / only one / who would / receive / the scholarship.
31. A group of employees were waiting for the new key distribution list.
    a. All the employees / expected that / [they/he] / would / likely / be the / only one / who would / get / the key.
    b. Every employee / expected that / [they/he] / would / likely / be the / only one / who would / get / the key.
    c. Each employee / expected that / [they/he] / would / likely / be the / only one / who would / get / the key.
32. At the high school a bunch of athletes were discussing the upcoming track meet.
    a. All the athletes / said that / [they/he] / would / surely / be the / only one / who could / win / multiple events.
    b. Every athlete / said that / [they/he] / would / surely / be the / only one / who could / win / multiple events.

c. Each athlete / said that / [they/he] / would / surely / be the / only one / who could / win / multiple events.

33. A bunch of gardeners were selling produce at the local market.
    a. All the gardeners / said that / [they/he] / would / likely / be the / only one / who would / make / lots of money.
    b. Every gardener / said that / [they/he] / would / likely / be the / only one / who would / make / lots of money.
    c. Each gardener / said that / [they/he] / would / likely / be the / only one / who would / make / lots of money.
34. A group of senators were drafting up new legislation.
    a. All the senators / said that / [they/he] / would / definitely / be the / only one / who would / propose / a popular bill.
    b. Every senator / said that / [they/he] / would / definitely / be the / only one / who would / propose / a popular bill.
    c. Each senator / said that / [they/he] / would / definitely / be the / only one / who would / propose / a popular bill.
35. A group of optometrists were gathered at the university.
    a. All the optometrists / expected that / [they/he] / would / likely / be the / only one / who was / wearing / new glasses.
    b. Every optometrist / expected that / [they/he] / would / likely / be the / only one / who was / wearing / new glasses.
    c. Each optometrist / expected that / [they/he] / would / likely / be the / only one / who was / wearing / new glasses.
36. A group of soloists were waiting for feedback.
    a. All the soloists / hoped that / [they/he] / would / ultimately / be the / only one / who would / receive / high praise.
    b. Every soloist / hoped that / [they/he] / would / ultimately / be the / only one / who would / receive / high praise.
    c. Each soloist / hoped that / [they/he] / would / ultimately / be the / only one / who would / receive / high praise.

4 EXPERIMENT 4 TEST MATERIALS: SELF-PACED READING, GENDERED ANTECEDENT

1. Outside the school, there was a group of boys talking about how the exam went.
    a. The boys / thought that / [they/he] / would / definitely / be the / only one / who is / going to get / an A plus.
    b. All the boys / thought that / [they/he] / would / definitely / be the / only one / who is / going to get / an A plus.
    c. Each boy / thought that / [they/he] / would / definitely / be the / only one / who is / going to get / an A plus.
2. At the fire hall early in the morning, a bunch of firemen were bragging about an upcoming competition.

a. The firemen / said that / [they/he] / would / certainly / be the / only one / who is / going to beat / the hall record.

b. All the firemen / said that / [they/he] / would / certainly / be the / only one / who is / going to beat / the hall record.

c. Each fireman / said that / [they/he] / would / certainly / be the / only one / who is / going to beat / the hall record.

3. After the restaurant closed for the night, a group of busboys were talking.

   a. The busboys / thought that / [they/he] / would / probably / be the / only one / who is / going to get / bad tips.

   b. All the busboys / thought that / [they/he] / would / probably / be the / only one / who is / going to get / bad tips.

   c. Each busboy / thought that / [they/he] / would / probably / be the / only one / who is / going to get / bad tips.

4. A group of men had applied for a prestigious scholarship at the university.

   a. The men / believed that / [they/he] / would / rightfully / be the / only one / who is / going to get / the scholarship.

   b. All the men / believed that / [they/he] / would / rightfully / be the / only one / who is / going to get / the scholarship.

   c. Each man / believed that / [they/he] / would / rightfully / be the / only one / who is / going to get / the scholarship.

5. A bunch of fathers were competing to make the best cake on a popular baking show.

   a. The fathers / hoped that / [they/he] / might / fortunately / be the / only one / who is / going to use / chocolate frosting.

   b. All the fathers / hoped that / [they/he] / might / fortunately / be the / only one / who is / going to use / chocolate frosting.

   c. Each father / hoped that / [they/he] / might / fortunately / be the / only one / who is / going to use / chocolate frosting.

6. A group of guys were deciding on a video game to play at the house party.

   a. The guys / believed that / [they/he] / would / surprisingly / be the / only one / who is / going to reject / Mario Kart.

   b. All the guys / believed that / [they/he] / would / surprisingly / be the / only one / who is / going to reject / Mario Kart.

   c. Each guy / believed that / [they/he] / would / surprisingly / be the / only one / who is / going to reject / Mario Kart.

7. A big group of cowboys decided to go for a ride despite the poor weather.

   a. The cowboys / thought that / [they/he] / would / surely / be the / only one / who is / going to like / the rain.

   b. All the cowboys / thought that / [they/he] / would / surely / be the / only one / who is / going to like / the rain.

   c. Each cowboy / thought that / [they/he] / would / surely / be the / only one / who is / going to like / the rain.

8. A group of strongmen were competing in the final of the weightlifting championship.
   a. The strongmen / thought that / [they/he] / would / naturally / be the / only one / who is / going to break / the world record.
   b. All the strongmen / thought that / [they/he] / would / naturally / be the / only one / who is / going to break / the world record.
   c. Each strongman / thought that / [they/he] / would / naturally / be the / only one / who is / going to break / the world record.
9. A bunch of schoolboys made it to the TV showing of the national Spelling Bee.
   a. The schoolboys / thought that / [they/he] / might / ultimately / be the / only one / who is / going to spell / the hardest word.
   b. All the schoolboys / thought that / [they/he] / might / ultimately / be the / only one / who is / going to spell / the hardest word.
   c. Each schoolboy / thought that / [they/he] / might / ultimately / be the / only one / who is / going to spell / the hardest word.
10. A group of handymen had applied for one permanent position at the new shop.
    a. The handymen / expected that / [they/he] / would / definitely / be the / only one / who is / going to get / the job offer.
    b. All the handymen / expected that / [they/he] / would / definitely / be the / only one / who is / going to get / the job offer.
    c. Each handyman / expected that / [they/he] / would / definitely / be the / only one / who is / going to get / the job offer.
11. A group of workmen offered to work during the Thanksgiving holiday.
    a. The workmen / thought that / [they/he] / would / naturally / be the / only one / who is / going to want / that shift.
    b. All the workmen / thought that / [they/he] / would / naturally / be the / only one / who is / going to want / that shift.
    c. Each workman / thought that / [they/he] / would / naturally / be the / only one / who is / going to want / that shift.
12. A group of salesmen were competing to win a prestigious award.
    a. The salesmen / hoped that / [they/he] / might / actually / be the / only one / who is / going to win / the award.
    b. All the salesmen / hoped that / [they/he] / might / actually / be the / only one / who is / going to win / the award.
    c. Each salesman / hoped that / [they/he] / might / actually / be the / only one / who is / going to win / the award.
13. A group of businessmen were waiting on the train platform at rush hour.
    a. The businessmen / thought that / [they/he] / would / fortunately / be the / only one / who is / going to get / a seat.
    b. All the businessmen / thought that / [they/he] / would / fortunately / be the / only one / who is / going to get / a seat.

   c. Each businessman / thought that / [they/he] / would / fortunately / be the / only one / who is / going to get / a seat.
14. A bunch of dads turned out early to see the parade through the city.
   a. The dads / thought that / [they/he] / would / definitely / be the / only one / who is / going to get / a good view.
   b. All the dads / thought that / [they/he] / would / definitely / be the / only one / who is / going to get / a good view.
   c. Each dad / thought that / [they/he] / would / definitely / be the / only one / who is / going to get / a good view.
15. At the park, a group of grandfathers met to discuss gardening for next year.
   a. The grandfathers / thought that / [they/he] / would / likely / be the / only one / who is / going to plant / more tulips.
   b. All the grandfathers / thought that / [they/he] / would / likely / be the / only one / who is / going to plant / more tulips.
   c. Each grandfather / thought that / [they/he] / would / likely / be the / only one / who is / going to plant / more tulips.
16. A group of brothers rushed to get an exclusive discount on a video game.
   a. The brothers / thought that / [they/he] / might / possibly / be the / only one / who is / going to get / the video game.
   b. All the brothers / thought that / [they/he] / might / possibly / be the / only one / who is / going to get / the video game.
   c. Each brother / thought that / [they/he] / might / possibly / be the / only one / who is / going to get / the video game.
17. A group of sons hoped to take over the autoshop.
   a. The sons / thought that / [they/he] / would / likely / be the / only one / who is / going to get / the autoshop.
   b. All the sons / thought that / [they/he] / would / likely / be the / only one / who is / going to get / the autoshop.
   c. Each son / thought that / [they/he] / would / likely / be the / only one / who is / going to get / the autoshop.
18. A group of baritones sang at a live concert to win a prize.
   a. The baritones / thought that / [they/he] / would / probably / be the / only one / who is / going to get / a prize.
   b. All the baritones / thought that / [they/he] / would / probably / be the / only one / who is / going to get / a prize.
   c. Each baritone / thought that / [they/he] / would / probably / be the / only one / who is / going to get / a prize.
19. At a family reunion, a group of uncles were competing in a sack race.
   a. The uncles / thought that / [they/he] / would / surely / be the / only one / who is / going to win / the race.

b. All the uncles / thought that / [they/he] / would / surely / be the / only one / who is / going to win / the race.

c. Each uncle / thought that / [they/he] / would / surely / be the / only one / who is / going to win / the race.

20. A group of linesmen had bet on the championship game.

a. The linesmen / thought that / [they/he] / might / ultimately / be the / only one / who is / going to guess / the winners.

b. All the linesmen / thought that / [they/he] / might / ultimately / be the / only one / who is / going to guess / the winners.

c. Each linesman / thought that / [they/he] / might / ultimately / be the / only one / who is / going to guess / the winners.

21. A group of cameramen had submitted work to a local festival.

a. The cameramen / expected that / [they/he] / would / actually / be the / only one / who is / going to attend / the festival.

b. All the cameramen / expected that / [they/he] / would / actually / be the / only one / who is / going to attend / the festival.

c. Each cameraman / expected that / [they/he] / would / actually / be the / only one / who is / going to attend / the festival.

22. At a conference, a group of weathermen were discussing the latest climate change research.

a. The weathermen / said that / [they/he] / would / certainly / be the / only one / who is / going to understand / the research.

b. All the weathermen / said that / [they/he] / would / certainly / be the / only one / who is / going to understand / the research.

c. Each weatherman / said that / [they/he] / would / certainly / be the / only one / who is / going to understand / the research.

23. A bunch of choirboys were having a disagreement about harmonizing.

a. The choirboys / said that / [they/he] / would / probably / be the / only one / who is / going to produce / the right note.

b. All the choirboys / said that / [they/he] / would / probably / be the / only one / who is / going to produce / the right note.

c. Each choirboy / said that / [they/he] / would / probably / be the / only one / who is / going to produce / the right note.

24. A group of fishermen were bragging about last summer.

a. The fishermen / thought that / [they/he] / might / possibly / be the / only one / who is / going to tell / an honest story.

b. All the fishermen / thought that / [they/he] / might / possibly / be the / only one / who is / going to tell / an honest story.

c. Each fisherman / thought that / [they/he] / might / possibly / be the / only one / who is / going to tell / an honest story.

25. A group of policemen were impressed with the new car for the department.

a. The policemen / believed that / [they/he] / would / actually / be the / only one / who is / going to drive / the new car.
b. All the policemen / believed that / [they/he] / would / actually / be the / only one / who is / going to drive / the new car.
c. Each policeman / believed that / [they/he] / would / actually / be the / only one / who is / going to drive / the new car.

26. At Christmas dinner, a bunch of nephews were at the table waiting for dessert.
    a. The nephews / believed that / [they/he] / would / probably / be the / only one / who is / going to get / a massive portion.
    b. All the nephews / believed that / [they/he] / would / probably / be the / only one / who is / going to get / a massive portion.
    c. Each nephew / believed that / [they/he] / would / probably / be the / only one / who is / going to get / an massive portion.
27. A group of lords were talking at the castle.
    a. The lords / said that / [they/he] / might / actually / be the / only one / who is / going to find / a good servant.
    b. All the lords / said that / [they/he] / might / actually / be the / only one / who is / going to find / a good servant.
    c. Each lord / said that / [they/he] / might / actually / be the / only one / who is / going to find / a good servant.
28. A group of mailmen were arguing about how to get downtown.
    a. The mailmen / thought that / [they/he] / would / definitely / be the / only one / who is / going to find / the fastest route.
    b. All the mailmen / thought that / [they/he] / would / definitely / be the / only one / who is / going to find / the fastest route.
    c. Each mailman / thought that / [they/he] / would / definitely / be the / only one / who is / going to find / the fastest route.
29. At the orchard, a group of farm boys were competing to pick the most fruit.
    a. The farm boys / thought that / [they/he] / would / surely / be the / only one / who is / going to fill / a dozen baskets.
    b. All the farm boys / thought that / [they/he] / would / surely / be the / only one / who is / going to fill / a dozen baskets.
    c. Each farm boy / thought that / [they/he] / would / surely / be the / only one / who is / going to fill / a dozen baskets.
30. A group of young dukes were discussing a large inheritance of land.
    a. The dukes / believed that / [they/he] / would / surely / be the / only one / who is / going to receive / the inheritance.
    b. All the dukes / believed that / [they/he] / would / surely / be the / only one / who is / going to receive / the inheritance.
    c. Each duke / believed that / [they/he] / would / surely / be the / only one / who is / going to receive / the inheritance.

31. A group of paperboys were waiting to hear about the new paper route.
    a. The paperboys / expected that / [they/he] / would / likely / be the / only one / who is / going to get / the paper route.
    b. All the paperboys / expected that / [they/he] / would / likely / be the / only one / who is / going to get / the paper route.
    c. Each paperboy / expected that / [they/he] / would / likely / be the / only one / who is / going to get / the paper route.
32. At the high school, a bunch of defensemen were discussing the upcoming soccer game.
    a. The defensemen / said that / [they/he] / would / surely / be the / only one / who is / going to score / multiple goals.
    b. All the defensemen / said that / [they/he] / would / surely / be the / only one / who is / going to score / multiple goals.
    c. Each defenseman / said that / [they/he] / would / surely / be the / only one / who is / going to score / multiple goals.
33. A bunch of priests were planning the Christmas Eve service.
    a. The priests / said that / [they/he] / would / likely / be the / only one / who is / going to say / the opening prayer.
    b. All the priests / said that / [they/he] / would / likely / be the / only one / who is / going to say / the opening prayer.
    c. Each priest / said that / [they/he] / would / likely / be the / only one / who is / going to say / the opening prayer.
34. A group of kings were having a discussion about a new peace treaty.
    a. The kings / said that / [they/he] / would / definitely / be the / only one / who is / going to determine / the final terms.
    b. All the kings / said that / [they/he] / would / definitely / be the / only one / who is / going to determine / the final terms.
    c. Each king / said that / [they/he] / would / definitely / be the / only one / who is / going to determine / the final terms.
35. A group of bachelors were gathered at a reunion at the university.
    a. The bachelors / expected that / [they/he] / would / likely / be the / only one / who is / going to get / engaged to be married.
    b. All the bachelors / expected that / [they/he] / would / likely / be the / only one / who is / going to get / engaged to be married.
    c. Each bachelor / expected that / [they/he] / would / likely / be the / only one / who is / going to get / engaged to be married.
36. A group of stuntmen were waiting for feedback.
    a. The stuntmen / hoped that / [they/he] / would / ultimately / be the / only one / who is / going to receive / high praise.
    b. All the stuntmen / hoped that / [they/he] / would / ultimately / be the / only one / who is / going to receive / high praise.

c. Each stuntman / hoped that / [they/he] / would / ultimately / be the / only one / who is / going to receive / high praise.
